# Epigenomic profiling of retinal progenitors reveals LHX2 is required for developmental regulation of open chromatin

**DOI:** 10.1038/s42003-019-0375-9

**Published:** 2019-04-25

**Authors:** Cristina Zibetti, Sheng Liu, Jun Wan, Jiang Qian, Seth Blackshaw

**Affiliations:** 1 Solomon H. Snyder Department of Neuroscience, Johns Hopkins University School of Medicine, Baltimore, MD 21205 USA; 2 Department of Ophthalmology, Johns Hopkins University School of Medicine, Baltimore, MD 21205 USA; 3 Department of Medical and Molecular Genetics, Indiana University School of Medicine, Indianapolis, IN 46202 USA; 4 The Sidney Kimmel Comprehensive Cancer Center, Johns Hopkins University School of Medicine, Baltimore, MD 21205 USA; 5 Department of Neurology, Johns Hopkins University School of Medicine, Baltimore, MD 21205 USA; 6 Center for Human Systems Biology, Johns Hopkins University School of Medicine, Baltimore, MD 21205 USA; 7 Institute for Cell Engineering, Johns Hopkins University School of Medicine, Baltimore, MD 21205 USA

**Keywords:** Epigenomics, Developmental neurogenesis

## Abstract

Retinal neurogenesis occurs through partially overlapping temporal windows, driven by concerted actions of transcription factors which, in turn, may contribute to the establishment of divergent genetic programs in the developing retina by coordinating variations in chromatin landscapes. Here we comprehensively profile murine retinal progenitors by integrating next generation sequencing methods and interrogate changes in chromatin accessibility at embryonic and post-natal stages. An unbiased search for motifs in open chromatin regions identifies putative factors involved in the developmental progression of the epigenome in retinal progenitor cells. Among these factors, the transcription factor LHX2 exhibits a developmentally regulated cis-regulatory repertoire and stage-dependent motif instances. Using loss-of-function assays, we determine LHX2 coordinates variations in chromatin accessibility, by competition for nucleosome occupancy and secondary regulation of candidate pioneer factors.

## Abstract (web summary)

Cristina Zibetti et al. present ATAC-seq, RNA-seq and ChIP-seq analyses of murine retinal progenitors at early and late developmental stages. They find a central role for the LIM homeodomain transcription factor LHX2 in the global control of chromatin accessibility and identify Lhx2-dependent candidate pioneer factors that may contribute to the establishment and maintenance of open chromatin in the retina.

## Introduction

Retinal neurogenesis occurs through partially overlapping temporal windows giving rise to individual cell types, as result of temporal patterning^1,2^ and context-dependent regulatory functions governed by transcription factors (TFs)^3–6^. A subset of transcription factors, commonly referred to as pioneer factors, occupy compacted chromatin regions to initiate or mediate chromatin opening, enabling nucleosomal accessibility to the transcriptional apparatus and instructing recruitment of accessory factors to reprogram cellular identity.

Even though the expression patterns have been extensively characterized for most of the retinal transcription factors, the mechanisms by which these coordinate epigenetic changes across retinal development remain elusive, as they are limited to select cases and often perturbed by experimental biases intrinsic to cell cultures systems and specific genetic background.

The LIM homeodomain transcription factor LHX2 has been characterized in a broad range of developmental contexts and it is required for the eye field specification and the morphogenesis of the optic cup through cell autonomous regulation of gene expression^7^. While *Lhx2* mutants are anophthalmic, display hypoplasia of the neocortex and severe anemia, neuroretina specific loss-of-function of *Lhx2* causes severe microphthalmia, loss of expression of a subset of retinal progenitor cells (RPC)-specific genes and ectopic expression of hypothalamic genes^8^.

Ocular expression of *Lhx2* has been observed as early as embryonic day e8.5, it can be detected in the retinal neuro-epithelium and retinal pigmented epithelium at e10 and ciliary margin by e14, becoming progressively restricted to the inner nuclear layer. In the post natal retina, LHX2 is expressed in mitotic retinal progenitor cells that co-express VSX2 and mKi67. By post natal day P7, LHX2 expression becomes restricted to differentiating Müller glia and is preserved in adult Müller glia, a subset of bipolar and starburst amacrine cells^9^. Early embryonic deletion of *Lhx2* results in proliferative defects of glial committed precursors while early post natal ablation, as well as overexpression^9^ results in the loss of glial markers and dysmorphic apical structures.

While temporally controlled conditional knockouts indicate that LHX2 induces and stabilizes the retinal glial fate during first post natal week of development, by sustaining the expression of the *Hes5*-mediated *Notch* signaling effectors^9,10^, its function does not seem to be confined to the differentiation of glial precursors: early embryonic ablation of *Lhx2* at e12.5 results in the depletion of multipotent retinal progenitors and overproduction of retinal ganglion cells (RGCs), while overproduction of rods is observed three days later. This suggests LHX2 may contribute to the maintenance of embryonic progenitors in a proliferative and multipotent state, by restricting their exit from the cell cycle and allowing for the generation of later cell types within discrete temporal windows^11^.

To investigate the mechanistic basis by which LHX2 orchestrates diverse developmental programs, we perform flow cytometry sorted analysis of dissociated retinal cells obtained from the neuro-retinal reporter *Chx10-Cre:eGFP* transgenic line^12^ that allows preferential retention of proliferating retinal progenitors at early and late stages of retinal development. We query the deriving epigenomic and transcriptional profiles obtained from natively extracted retinal progenitor cells for LHX2 representation and function over the course of retinal neurogenesis and we show LHX2 controls chromatin accessibility by modulating expression and co-opting action of developmentally regulated transcription factors with high pioneer potential.

## Results

**Fig. 1:**
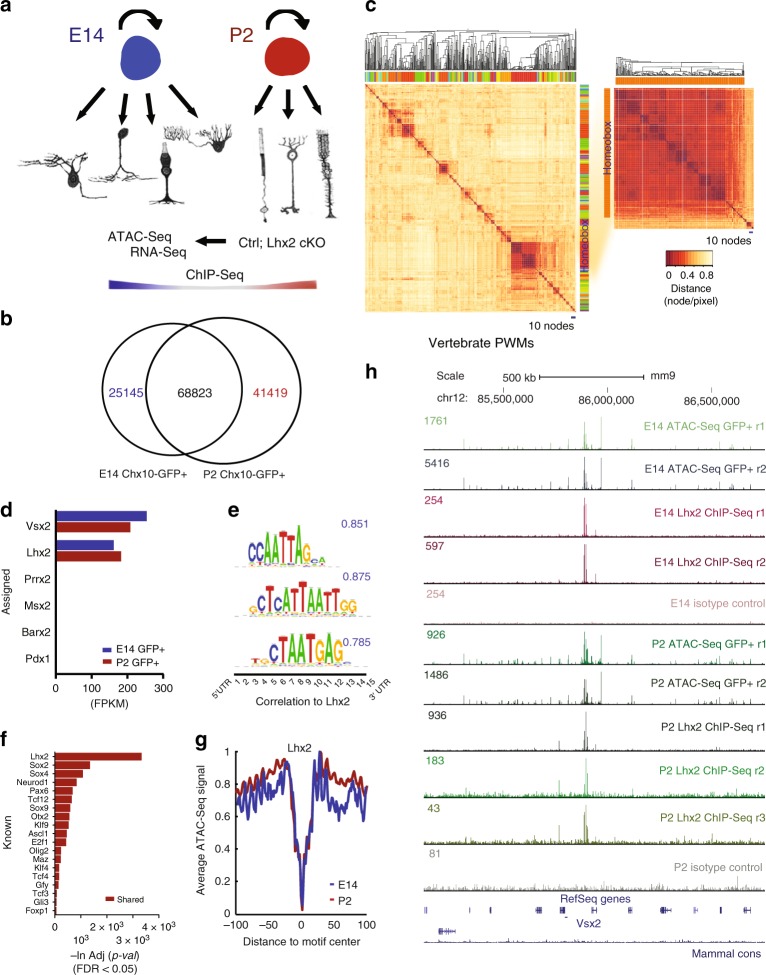
Pairwise comparison of ATAC-Seq data identifies open chromatin regions from early and late RPCs, with a broad overrepresentation of LHX2 related motifs. **a** Workflow for epigenomic profiling of RPC. **b** Venn-diagram represents open chromatin regions identified in early- and late-stage VSX2 (CHX10)—GFP-positive RPCs. **c** Hierarchical clustering of known motifs in the vertebrate genome (left, Jaspar 2016 non-redundant vertebrates core). Inset represents the major cluster of probabilistically assigned position weight matrices (PWMs) identified in open chromatin regions from early- and late-stage RPCs (scale = 1 node/pixel) (linkage = average; similarity threshold cor = 0.6, ncor = 0.4, *w* = 5) (Lhx2 instances as blue pixel strokes). **d** Relative RNA-Seq expression for homeobox transcription factors. **e** Representative LHX2 logos (MA0700.1, k-mer sig = 300, *e*-value = 1e-300), with positional variations of the same motif instance. **f** Known motifs enrichment in open chromatin regions from early and late RPCs by binomial scoring of PWMs. **g** LHX2 footprints from open chromatin regions identified in early- and late-stage RPCs. **h** Custom tracks of RPC-derived ATAC-Seq profiles and aged-matched LHX2 ChIP-Seq feature the *Vsx2* locus (mm9)

Fluorescence-activated cell sorting was adopted to isolate retinal progenitor cells from mice expressing the *Chx10-Cre:eGFP* transgene^12^ from embryonic day (E)14 and post natal day (P)2 retina, representing early and late stages of retinal progenitors competence, respectively^1^. The brightest fraction of GFP-positive cells expresses the retinal progenitor-specific markers VSX2 (*Chx10*), mKi67 and CCND1 and was separated from the dim and GFP-negative (Supplementary Fig. 1A–I). Proliferating retinal progenitor cells will be hereafter referred to as RPCs or GFP+ and the post mitotic pool as GFP−. Flow-sorted cells were profiled by RNA-Seq and ATAC-Seq, relying on direct in vitro transposition of sequencing adapters into native chromatin^13^. The preliminary screening of the ATAC-Seq-derived open chromatin regions identified LHX2 as top candidate transcription factor. ChIP-Seq was then performed on LHX2 and the related binding sites were integrated and compared with the age-matched RNA-Seq and ATAC-Seq profiles from control and knock-out retinas (Fig. 1a and Supplementary Fig. 1J, K).

### Open chromatin unbiased screening reveals top candidate TF

The ATAC-Seq-derived open chromatin regions were identified by pairwise comparison of normalized read counts from VSX2 (*Chx10*)-positive cell fractions at E14 and P2 and assessed for reproducibility (Supplementary Fig. 2A–D). The open chromatin regions shared between time points represent 2.4% of the genome and those specific to either E14 or P2 RPCs represent 0.72 and 0.80% of the genome, respectively (Fig. 1b).

**Fig. 2:**
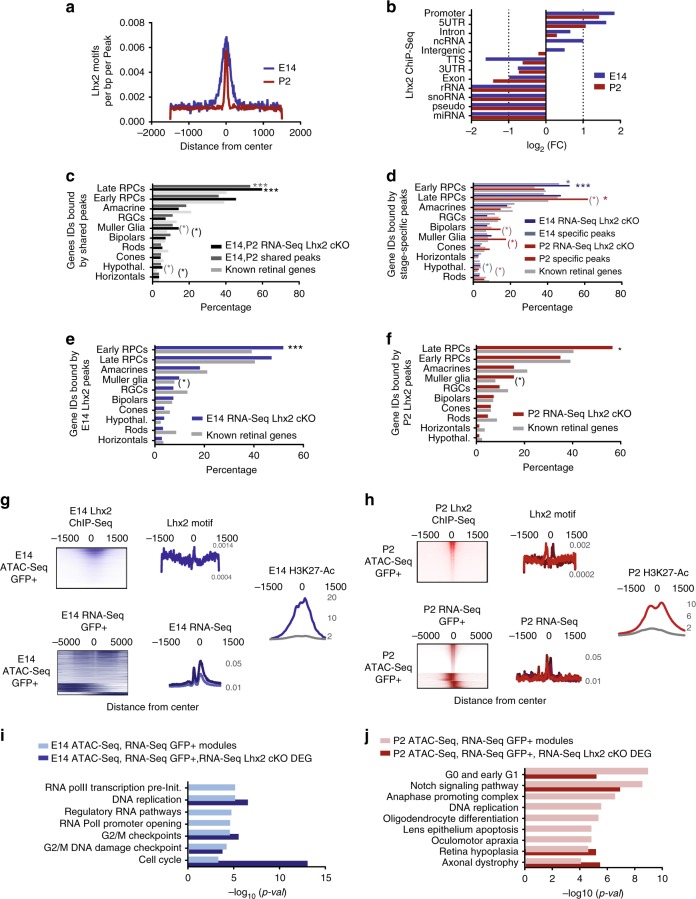
LHX2 regulates cell cycle genes and the Notch signaling pathway by targeting promoters and non-coding elements in nucleosome free regions associated with active enhancers. **a** LHX2 motif density around the centers of ChIP-Seq peaks at E14 and P2. **b** Enrichment of LHX2 ChIP-Seq peaks distributed across different genomic regions (log_2_ fold enrichment). **c**–**f** Percentage of LHX2 target genes in retinal term assigned to at least one age-matched LHX2 ChIP-Seq peak. Enrichment was computed between target genes in term and total number of known genes in term (Supplementary Table 4 and Supplementary Data 1) for peaks shared at E14 and P2 (**c**), stage-specific peaks (**d**), and all peaks detected at E14 (**e**) and P2 (**f**). RNA-Seq from age-matched *Lhx2* cKO retinas identifies *Lhx2*-dependent genes sets. (*p-value < 0.05, ***p-value < 0.001). Asterisks in parenthesis refer to *p*-values before Bonferroni–Hochberg correction (target genes populating the enriched ontologies are in Supplementary Data 2). **g**, **h** Heatmaps of raw reads from LHX2 ChIP-Seq peaks are plotted across nucleosome centered regions from age-matched ATAC-Seq samples. Each row represents a 3 kb window centered at maximum read pile-up. LHX2 motif occurrence in open chromatin regions is displayed and reported in Supplementary Tables 6. LHX2 motifs co-localize with RNA-Seq raw reads. Meta-profiles of the class II enhancer-associated H3K27ac marks were compiled at bidirectionally transcribed regions from the E14 (**g**) and P2 RPCs fractions (**h**) (background in gray, opposite strands replicates in hue). **i**, **j** Functional enrichment of *Lhx2*-dependent genes sets encoded within open chromatin regions (Supplementary Tables 6) by binomial distribution

We next scanned open chromatin regions common to E14 and P2 RPCs for enriched oligonucleotide motifs to identify candidate transcription factor binding sites. Hierarchical clustering of probabilistically assigned position weight matrices revealed that homeobox domain-containing transcription factor motifs constitute the prevalent cluster, representing 284 out of 373 putative matches (Fig. 1c), followed by C2H2- type, GC-rich and KLF zinc finger proteins, then POU-homeodomain, MADS box and CCAAT-Binding factors; individual motif instances were found associated to NFY factors and the insulator protein CTCF, involved in chromatin looping and conformation. Among the putatively assigned homeobox transcription factors for which a motif instance could be found, just few are expressed in RPCs (Fig. 1d) and many represented positional variations of the LHX2 consensus (Fig. 1e and Supplementary Fig. 2E). LHX2 enrichment was inferred by binomial scoring of known position weight matrices as the most represented transcription factor motif in open chromatin regions common to early and late RPCs (Fig. 1f and Supplementary Table 1), and variably represented in stage-specific accessible regions (Supplementary Tables 2 and 3). LHX2 occupancy resulted into detectable footprints at both stages (Fig. 1g). ChIP-Seq was performed for the top candidate transcription factor LHX2 in E14 and P2 mouse retina and integrated with age-matched RNA-Seq and ATAC-Seq profiles from flow-sorted RPCs (Figs. 1h and 2a and Supplementary Fig. 3A–G).

### Dynamic allocation of LHX2-binding sites across development

LHX2 ChIP-Seq-binding sites are predominantly located in intergenic and intronic regions of the genome (Supplementary Fig. 4A) and enriched in promoter regions, 5′UTR, introns and ncRNA at both time points (Fig. 2b). The associated genes are enriched for functions related to the control of retinal development (Supplementary Fig. 4B, C) and the binding sites exhibit developmental differences in chromatin accessibility (Supplementary Fig. 4D).

The LHX2 cis-regulatory repertoire (ChIP-Seq peaks) (Supplementary Fig. 3G) was annotated based on proximity to the closest TSS profiled by RNA-Seq from age-matched RPCs (Supplementary Table 4) and annotated after the manually curated lists of retinal cell-type specific/enriched genes (Supplementary Data 1). Gene Ontology enrichment was computed based on the number of LHX2 target genes in term, for which at least one nearest peak could be found, populating a given ontology (Fig. 2c–f).

In order to evaluate whether LHX2 directly impacts the retinal transcriptome, RNA-Seq transcriptional profiles were also obtained from E14 (*Chx10Cre;Lhx2*^*lox/lox*^ retinas) (Supplementary Fig. 5A–D) and P2 (*Lhx2* ^*lox/lox*^ retinas electroporated with pCAG-*Cre-GFP* at P0 and isolated by FACS) *Lhx2* cKO conditions (Supplementary Fig. 5E, F). Transcriptional dependence of a given retinal gene on LHX2, first inferred by ChIP-Seq peaks proximity to the closest TSS, was confirmed by comparison of LHX2 annotated Peaks with RNA-Seq from age-matched *Lhx2* cKO (Supplementary Data 2.1–2.3 and Supplementary Fig. 5G) and gene ontology enrichment analysis was repeated (Fig. 2c–f). LHX2 ChIP-Seq peaks shared between time points were preferentially associated with late-stage RPCs genes and, to a lesser extent, with Müller glia and anterodorsal hypothalamus (Fig. 2c), while peaks found only at E14 were selectively associated with genes expressed in early-stage RPCs (Fig. 2d). Conversely, late RPCs were overrepresented in the P2 ChIP-Seq dataset (Fig. 2d).

More than 70% of the amacrine and retinal ganglion cell-enriched genes were induced in the E14 *Lhx2* cKO retinas, followed by horizontals, bipolars and late RPCs related genes, while Müller glia, early RPCs and photoreceptors related genes were prevalently downregulated. Conversely, photoreceptor-enriched genes were prevalently induced in the P2 *Lhx2* cKO retinas while horizontals related genes were lost (Supplementary Fig. 5H and Supplementary Data 2.4). As a fraction of LHX2 ChIP-Seq-binding sites fall in chromatin regions that are not transcriptionally active in RPCs at P2 (Supplementary Fig. 4D), LHX2 may potentially repress retinal type specific targets within critical windows to prevent their ectopic expression, as suggested by previous knock-out studies^11^. In total, >30% of the LHX2 peaks shared between time points and 20% of the stage-specific peaks could be assigned by proximity to differentially expressed genes detected from the comparison between E14 and P2 RPCs, potentially accounting for 10–14% of the differentially expressed transcriptome. Most of the differentially expressed genes, as well as genes constitutively enriched in early and late RPCs compared to the corresponding post mitotic fraction, were associated with stage-specific LHX2 peaks (Supplementary Table 4). Importantly, 47% of all differentially expressed RPCs associated genes show dependence on *Lhx2* expression in at least one developmental time point exhibiting variations in expression upon *Lhx2* loss-of-function (Supplementary Fig. 5G, Supplementary Table 5, and Supplementary Data 2). This indicates a central role of LHX2 in the regulation of RPCs transcriptome.

### LHX2 binds within coordinately accessible chromatin modules

In total, 4.25% and 3.48% of open chromatin regions overlapped with age-matched LHX2 ChIP-Seq peaks identified at E14 and P2, respectively, and a higher fraction of closely co-localizing peaks was observed at E14 compared to P2 (Supplementary Table 6).

Notably, LHX2 ChIP-Seq peaks were preferentially co-localized with positioned nucleosomes at E14 (Fig. 2g) but bimodal at P2 (Fig. 2h), suggesting LHX2 may compete for nucleosomes binding in early RPCs. Overall, 9.87% of the nucleosomes interspersed open chromatin regions identified in E14 RPCs, and 9.25% of those identified at P2 are coupled with bidirectionally transcribed sites to which LHX2 binding is associated. These represent 19.27% of the high-confidence LHX2 ChIP-Seq peaks at E14 (0.76% co-localizing) and 17.37% at P2 (0.73% co-localizing). The subset of LHX2 targets that flanked positioned nucleosomes within open chromatin regions were enriched at 5′UTR and promoter regions at E14 (Fig. 2g, log_2_FE = 6.27, 5.45, respectively) and P2 (Fig. 2h log_2_FE = 6.33, 5.2). Furthermore, LHX2-binding sites were coupled with bimodally distributed H3K27ac profiles at P2, a characteristic of active enhancers. Exons and ncRNA were also found among the enriched categories (E14 log_2_FE = 2.05, 2; P2 log_2_FE = 2.28, 2.16, respectively), where co-localization of transcripts with H3K27ac meta-profiles may potentially underlie the biogenesis of enhancer-templated eRNAs. The LHX2 targets identified in the E14 RPCs open chromatin regions were involved in regulatory RNA pathways, promoter opening and cell cycle checkpoints (Fig. 2i) and those in P2 RPCs population were enriched for functional categories related cell cycle progression, *Notch* signaling and DNA replication, as well as phenotypes such as retina hypoplasia and axonal dystrophy (Fig. 2j). Enrichment for the related ontologies was confirmed by comparison with *Lhx2*-dependent gene sets, identified by RNA-Seq from the age-matched *Lhx2* cKO (Supplementary Table 6.2). Examples of such LHX2 regulated genes include the locus encoding *Ndnf*, upregulated in P2 *Lhx2* cKO cells and the late-stage RPC and Müller glia -enriched *Car2* locus, which is downregulated in P2 *Lhx2* cKO cells.

The majority of LHX2 target sites are transcriptionally accessible: 76.66% of the E14 LHX2 cis-regulatory sites fall in GFP-positive open chromatin regions and 61.72% of the P2 ChIP-Seq peaks co-occur with P2 GFP-positive open chromatin regions (Supplementary Table 7).

We next scanned LHX2 ChIP-Seq peaks from early and late retinal stages to infer transcription factors co-occurrences. Multiple transcription factor motif instances were found within a broad range of similarity to the known LHX2 consensus and exhibited differential occupancy, possibly underlying differences in affinity and/or combinatorial interaction with other co-factors (Supplementary Fig. 6A–I).

**Fig. 3:**
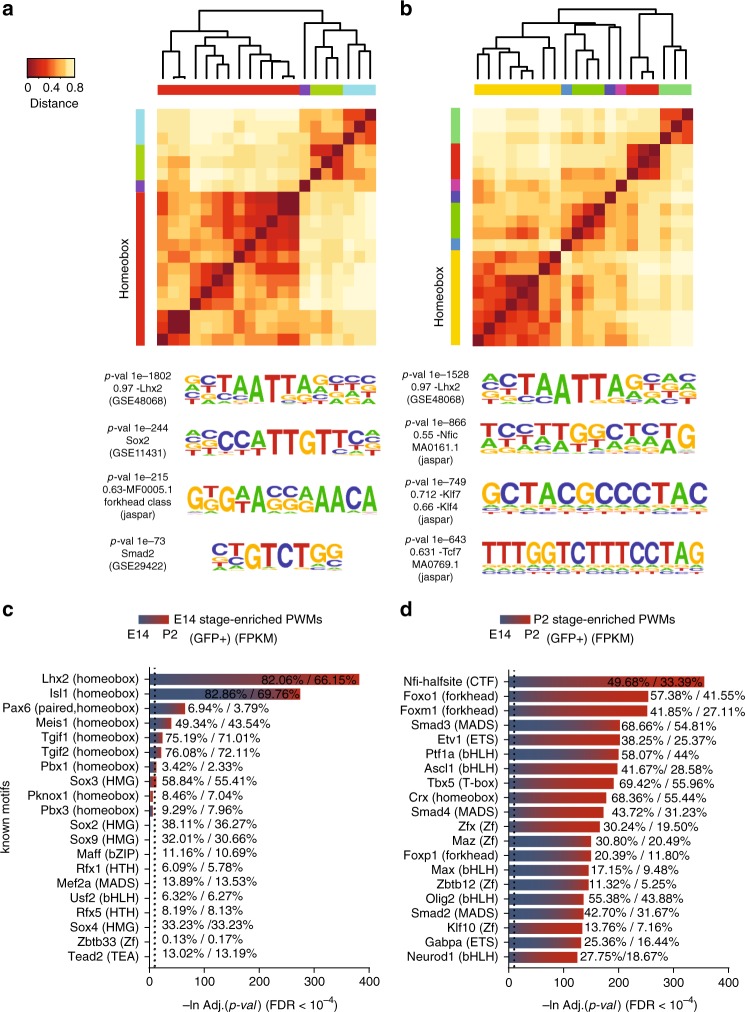
Binding sites for transcription factors with predicted pioneer function co-occur with LHX2 peaks. **a**, **b** Hierarchical clustering of LHX2 ChIP-Seq regulatory motifs and assigned representative logos are represented at E14 (**a**) and P2 (**b**) (linkage = average; similarity threshold cor = 0.6, ncor = 0.4, *w* = 5). The most enriched cluster comprises LHX2 and multiple variations of the same motif. **c**, **d** Known transcription factor motifs preferentially enriched in either E14 or P2 LHX2 ChIP-Seq peaks and identified by pairwise comparison are shown. Percentage of motif occurrences are reported for input and background datasets. The relative expression level of the corresponding transcription factor mRNA at E14/P2 is indicated by the blue/red color gradient, respectively

Novel LHX2 motifs were overrepresented within each ChIP-Seq dataset (Fig. 3a, b), followed by putative co-factors; some are Yamanaka factors, known to regulate the developmental signaling network in mouse embryonic stem cells^14^ and to exert a pioneer function in non-retinal developmental contexts^15,16^. The closest assignments were confirmed at each developmental stage by known motifs (Supplementary Fig. 7A) with preferential representation of homeobox/bHLH transcription factors at E14 (Fig. 3c) and CTF/NF-I and forkhead factors at P2 (Fig. 3d), although a wider variety was seen in the repertoire of P2 preferential motifs (Supplementary Fig. 7B). Specifically, among LHX2 co-occurrent motifs, the SoxB1 related family members (SOX2)^16^ and MADS transcription factors were found at E14 (Fig. 3a) while Kruppel like factors (KLF9/13)^14^, NF-I family members (NFIA/NFIB/NFIC/NFIX)^17^, SoxE (SOX8/9)^18^, and ASCL1^19^ were found at P2 (Fig. 3b).

### LHX2 regulates local and global chromatin accessibility

**Fig. 4:**
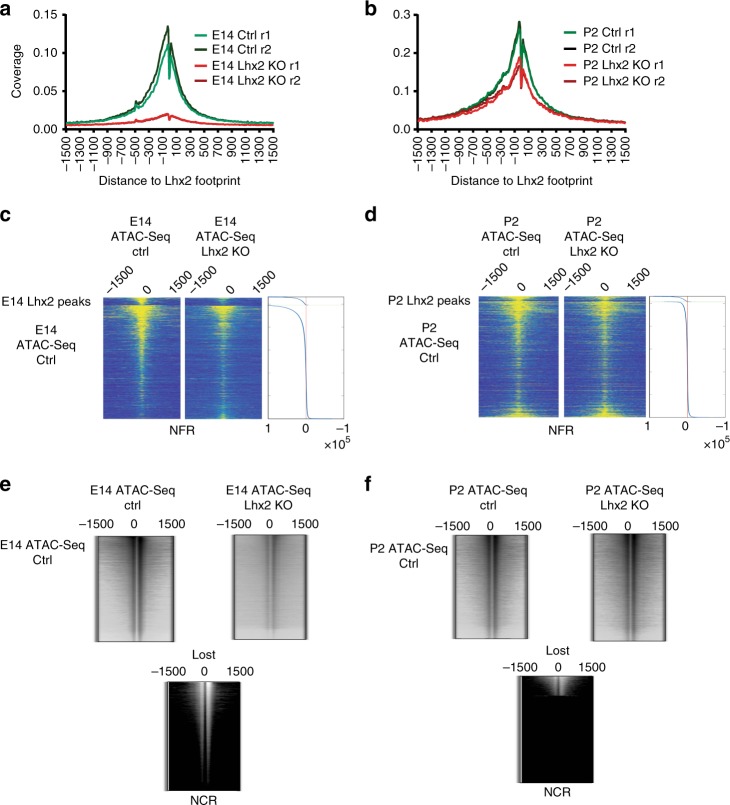
LHX2 affects local and global chromatin accessibility. **a**, **b** ATAC-Seq read distribution was plotted relative to the center of LHX2 footprints from controls and age-matched *Lhx2* cKO. **c**, **d** Read distribution profiles across nucleosome free regions (nfr) are derived from ATAC-Seq control and *Lhx2* cKO, plotted around age-matched LHX2 ChIP-Seq peaks centers and across all the identified age-matched open chromatin regions in a 3 kb window. The densitometric difference in read coverage between control and *Lhx2* cKO is reported on the right. **e**, **f** Read distribution profiles flanking nucleosomes centered regions (ncr) are derived from ATAC-Seq control and *Lhx2* cKO and plotted across all the identified age-matched open chromatin regions in a 3 kb window

To assess whether LHX2 regulates chromatin accessibility, we compared ATAC-Seq profiles from purified RPCs at E14 and P2 to those obtained from age-matched conditional knock-outs (Fig. 4a–f and Supplementary Fig. 8A–E). In E14 *Chx10Cre;Lhx2*^*lox/lox*^ retinas, the read coverage and footprint depth at LHX2 center were fourfold and eightfold reduced, respectively (Fig. 4a), while in P2 *Lhx2*^*lox/lox*^ retinas, a detectable, yet less pronounced reduction in the read coverage and footprint depth could be observed (Fig. 4b), possibly as a result of LHX2 perdurance in P0 electroporated cells, following CRE-mediated deletion.

**Fig. 5:**
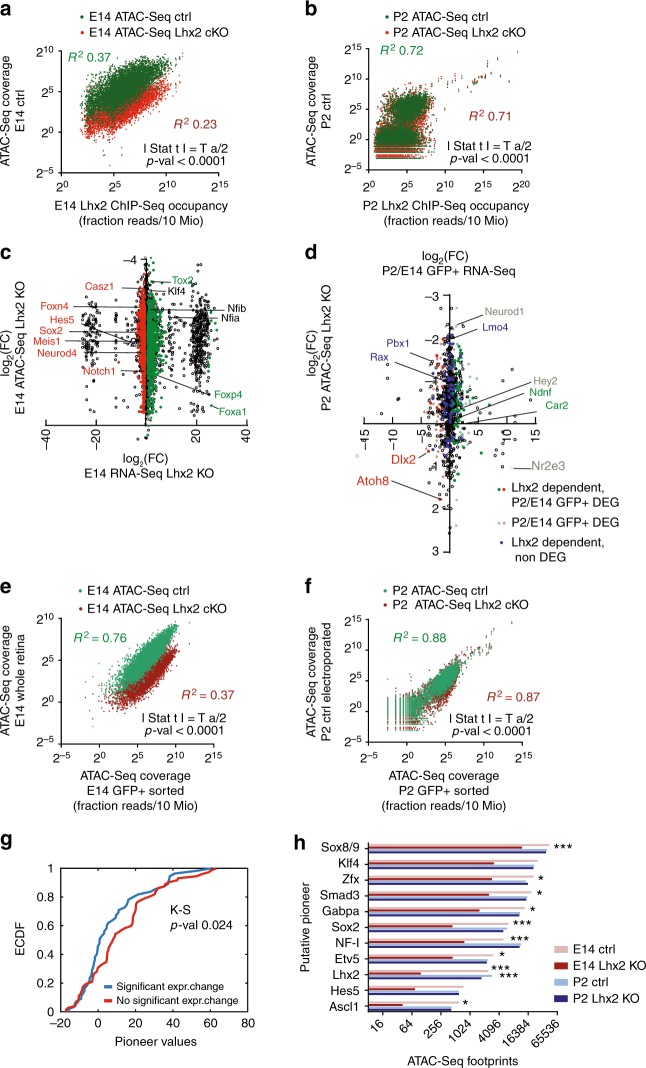
LHX2 affects local and global chromatin accessibility by epistatic and steric regulation of transcription factors with high pioneer potential. **a**, **b** Correlation between LHX2 occupancy and local accessibility at LHX2 target sites in age-matched open chromatin regions in E14 and P2 control conditions and in *Lhx2* cKO. Two-tailed *t*-test statistics is reported. For loss of accessibility statistics at LHX2 targets, refer to Supplementary Table 7. **c**, **d** Paired variations in gene expression and local chromatin accessibility are displayed for loci encoding LHX2 targeted transcription factors in embryonic *Lhx2* cKO (Supplementary Data 3) (**c**) and developmentally regulated post natal targets (**d**). Representative transcription factors are highlighted with arrows (green/red for up/downregulation by RNA-Seq). **e**, **f** Correlation of open chromatin profiles obtained from ATAC-Seq flow-sorted fractions from control and *Lhx2* cKO, normalized by the library size. Two-tailed *t*-test statistics is reported. For global loss of accessibility refer to Supplementary Table 8. **g** Cumulative distribution of differentially expressed transcription factors across intervals of pioneer potential. Transcription factors with predicted pioneer potential based on P2 RPCs accessibility profiles compared to E14 were identified and filtered based on RNA-Seq. Kolmogorov–Smirnov test was applied to putative pioneer TF with differential (blue) or comparable (red) expression between time points. Differentially expressed genes are distributed across higher intervals of pioneer potential than those shared between time points. **h** Footprint counts for individual transcription factors with predicted pioneer activity matching LHX2 co-occurrent motifs are shown in control and *Lhx2* cKO from E14 and P2 retina. For predicted pioneer potential, refer to Supplementary Table 12

Upon *Lhx2* loss-of-function variations in local accessibility occur at LHX2 target sites (Fig. 4c, d upper panel, Fig. 5a–d, and Supplementary Table 7) and a correlation between local accessibility and LHX2 occupancy was observed at both time points (Fig. 5a, b). Specifically, 8% of the LHX2 targets lose accessibility in *Lhx2* cKO at P2, while 40% of the LHX2 targets are affected at embryonic stages (Supplementary Table 7, and Supplementary Figs. 9 and 10), indicating LHX2 contributes to the maintenance of chromatin accessibility of its binding sites.

The effect of *Lhx2* loss-of-function on chromatin accessibility was not merely focal, but extended genome-wide. While ATAC-Seq profiles from E14 whole retina (Fig. 5e) and P2 electroporated control (Fig. 5f) showed high correlation with those from each corresponding flow-sorted RPCs fraction, overall, 50% of the globally profiled open chromatin regions are lost in E14 *Lhx2* cKO retinas and 22% are lost at P2 (Fig. 4c, d lower panel, Fig. 4e, f, and Supplementary Table 8), suggesting *Lhx2*-dependent mechanisms may concur to the global loss of chromatin accessibility. This may result from the regulation of chromatin remodeling factors and pioneer transcription factors that are expressed in the developing RPCs. Indeed, multiple LHX2 cis-regulatory elements that exhibit variation in local accessibility upon *Lhx2* loss-of-function and, that are assigned by proximity to *Lhx2-*dependent genes sets, encode for chromatin remodeling factors (Supplementary Table 6.2) and transcription factors with predicted pioneer function (Fig. 5c, d and Supplementary Table 9).

### *Lhx2*-dependent TFs pioneer chromatin opening

We next determined whether binding by known and putative pioneer factors expressed in RPCs occurs (Fig. 5g) and tested whether it was altered by loss of LHX2 (Supplementary Fig. 5H and Supplementary Tables 10 and 11), as result of transcriptional de-regulation and/or loss of steric interaction.

**Fig. 6:**
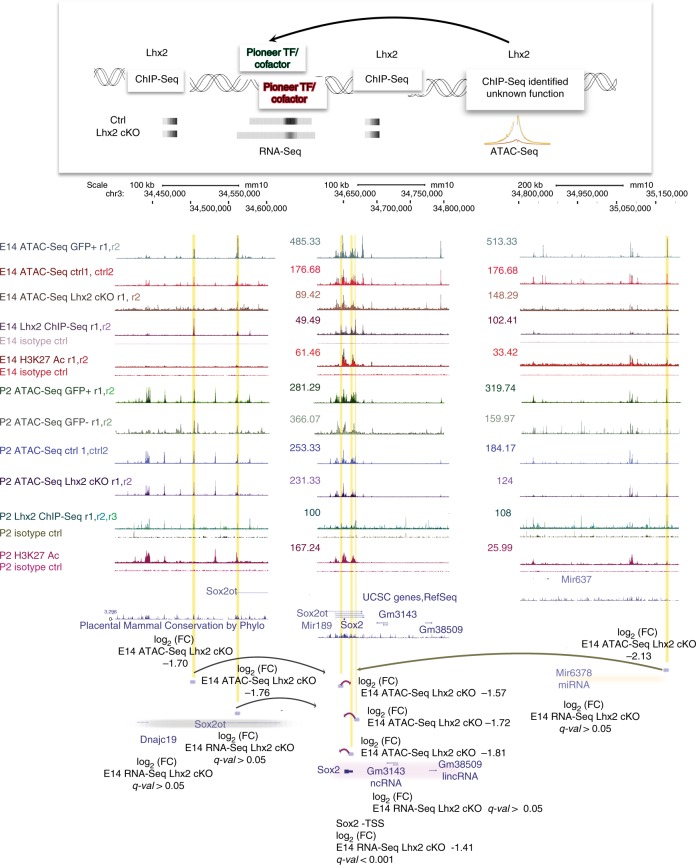
LHX2 coordinates variations in chromatin accessibility at the *Sox2* locus. Custom tracks of E14 and P2 LHX2 ChIP-Seq reads and age-matched ATAC-Seq from purified RPCs and post mitotic precursors were configured on the mm10 UCSC murine genome assembly. Regions of interest targeted by LHX2, where variations by RNA-Seq and/or ATAC-Seq coverage are observed in *Lhx2* cKO retinas are highlighted. Association with H3K27ac-labeled enhancer elements is also indicated. The LHX2-coordinated regulatory module is defined as follows: regulatory regions that display variations in ATAC-Seq coverage without a corresponding variation in the nearest transcript by E14 *Lhx2* cKO RNA-Seq are putatively assigned (arrows) to the nearest gene exhibiting variation in transcript levels

Some retinal progenitor-expressed transcription factors, whose motifs are predicted to co-occur with LHX2 peaks, (Fig. 3c, d and Supplementary Fig. 7A, B) have been previously characterized for their pioneer function in non-retinal contexts, such as KLF (KLFf9)^13^, SoxB1 family members^15^, and NF-I (NFIA/NFIB/NFIC/NFIX)^16^. *Sox2* is expressed in early and late-stage RPCs and is also a transcriptional target of LHX2 (Fig. 5c) for which coordinated variations in local accessibility are found within a range of 500 Kb from the TSS (Fig. 6).

**Fig. 7:**
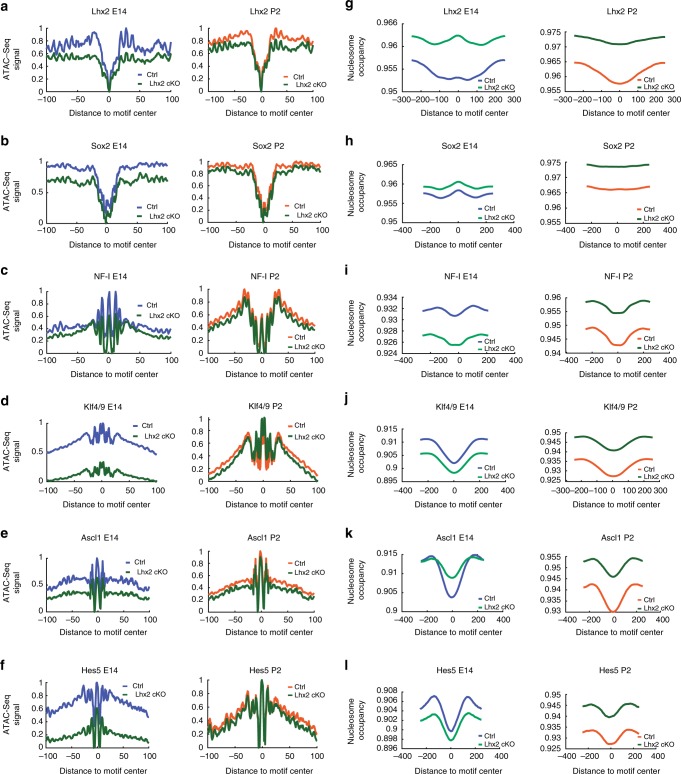
Footprinting analysis and competition for nucleosome occupancy by predicted pioneer factors reflect their developmentally regulated reliance on LHX2. **a–f** ATAC-Seq average cut profiles showing footprints for LHX2, SOX2, NF-I (NFIA/B/X), KLF4/9, ASCL1, and HES5 from E14 and P2 control and *Lhx2* cKO samples. **g–l** Nucleosome occupancy at motif centers is reported for LHX2, SOX2, NF-I, FIA/B/X), KLF4/9, ASCL1, and HES5 in E14 and P2 control and *Lhx2* cKO samples

First, we computed the probability of occupancy for candidate transcription factor binding sites in the genome to which position weight matrices can be assigned and where focal depletion of the ATAC-Seq reads is observed (footprints). We then estimated transcription factor-specific chromatin opening indexes from ATAC-Seq developmental time points^15^ at P2 compared to E14. Finally, we iterated the analysis in the *Lhx2* cKO conditions. Hence, we predicted recruitment of putative pioneer factors, by taking into account transcription factors expression patterns and querying ATAC-Seq profiles for detectable footprints (Fig. 7a–f), chromatin accessibility profiles and derivative nucleosome occupancy (Fig. 7g–l) in RPCs at E14 and P2.

In E14 *Chx10Cre;Lhx2*^*lox/lox*^ retinas, the average cut frequency at LHX2 motif center was reduced and in P2 *Lhx2*^*lox/lox*^ retinas a minor reduction in the average cut frequency (Fig. 7a) was also observed. Overall, 96% of LHX2 footprints from E14 RPCs were lost in E14 *Lhx2* cKO retinas and 39% were lost at P2 (Supplementary Tables 10 and 11). Footprints associated with SOX2, NF-I, SOX8/9 and ASCL1^16–19^ were statistically reduced in E14 *Lhx2* cKO (Fig. 5h), indicating that the recruitment of these transcription factors was altered following loss of LHX2. SOX2 and NF-I exhibited a 93 and 84% reduction in the footprints count (Supplementary Table 10), together with KLF4, ASCL1, and HES5; SOX2 and NF-I had altered mean scores (Supplementary Table 11) and meta-profiles around motif centers (Fig. 7b, c). Variations in mean scores and footprinting meta-profiles were also observed for the predicted pioneer KLF4/9, as well as ASCL1 and HES5 (Fig. 7d–f), known neurogenic and gliogenic factors respectively^19,20^, expressed in late-stage RPCs^21,22^. Other transcription factors that exhibit a high pioneer potential in the retina were transcriptionally targeted by LHX2 (Fig. 5c, d) and/or predicted to interact with LHX2, such as SOX9, SMAD3, ZFX, and ETV5, because variations in recruitment at active sites (Supplementary Table 11) and in the average footprint count (Fig. 5h and Supplementary Table 12) were observed upon *Lhx2* loss-of-function.

Nucleosome occupancy at the aforementioned motifs was altered in *Lhx2* cKO samples (Fig. 7g–l), suggesting that LHX2 may facilitate or stabilize recruitment of cognate factors on chromatin regions, prior to or upon chromatin opening. LHX2 seemed to compete for nucleosome occupancy (Fig. 7g), as its meta-profiles were reminiscent of those previously described for the non-canonical pioneer factor NFIB^17^. In E14 and P2 *Lhx2* cKO retinas, an overall increase in nucleosomes coverage occurs around LHX2 motif center, consistent with a reduction in chromatin accessibility observed upon *Lhx2* loss-of-function. Similarly, nucleosome depletion was observed at ASCL1 motif center in control conditions. Conversely, chromatin compaction occurs following *Lhx2* loss-of-function, as seen from rescued levels of nucleosome occupancy (Fig. 7k). Moreover, we noticed a global reduction in nucleosome occupancy 500 bp around NF-I, KLF4/9, and HES5 motif centers in E14 *Lhx2* cKO retinas and observed the opposite effect at post natal stages. No difference in competition for nucleosome binding could be noticed directly on NF-1 and KLF4/9 motif centers following *Lhx2* loss-of-function (Fig. 7i, j, l). Chromatin compaction also occurs around SOX2 motif center in the *Lhx2* cKO (Fig. 7h), with dips in nucleosomes distribution flanking SOX2 motif centers, suggesting SOX2 may preferentially be recruited on nucleosome free regions at E14, potentially acting as a settler factor whose binding occurs in proximity to pre-existing open chromatin states, as established by pioneer factors^15^.

## Discussion

In this study, we report a central role for LHX2 in the global control of chromatin accessibility in retinal progenitor cells, we identify possible mechanisms by which LHX2 may mediate retinal transcriptional networks across development and we provide what is, to our knowledge, the first systematic query of candidate pioneer factors that may contribute to the establishment and maintenance of open chromatin states in the native context of the developing murine retina.

First, an unbiased search for transcription factor motifs enriched in accessible chromatin regions from early and late retinal progenitor cells identified LHX2 as the most represented canonical transcription factor. LHX2 cis-regulatory repertoire changes over time, where the selective occupancy of retinal targets is reflected into gene variations upon *Lhx2* loss-of-function. At embryonic stages, LHX2 preferentially targets known early retinal progenitor-related genes while, at post natal stages, LHX2 targets known late progenitor-related genes. Divergence of LHX2 motif instances from the consensus results in developmentally regulated occupancy and may possibly underlie preferential targeting of retinal type specific targets in RPCs.

Individual overexpression of the Sry-related high-mobility group (HMG) transcription factors SOX2, SOX8, and SOX9 in the post natal retina has been shown to promote amacrine cells formation in *Lhx2* cKO mice, but not in wild-type animals. Likewise, electroporation of the *Notch* intracellular domain N1ICD in *Lhx2* cKO mice inhibits RPCs maintenance while promoting the expression of gliogenic markers, yet the phenotype was not observed electroporating the wild type retina^10^, suggesting LHX2 may deflect default developmental pathways by combinatorial interaction with co-factors, such as SOX9, restraining their ability to give rise to cellular diversity.

While LHX2 has a prominent role in the regulation of the transcriptome of early progenitors, a more moderate effect can be seen at post natal stages. The lower extent to which *Lhx2* loss-of-function affects gene expression at post natal stages may be, in part, result of LHX2 residual expression in neonatal electroporated cells, where the perdurance of the GFP reporter in progenitors exiting the cell cycle after transfection may contribute to the profiles analyzed. However, differential motif enrichment analysis indicates the post natal retinal transcriptome relies on a broader set of transcriptional networks, which may compensate for *Lhx2* ablation, limiting the magnitude of the transcriptional variations. This would be particularly relevant in the post natal retina as opposed to the embryonic knock-out, as the endogenous expression of LHX2 that precedes its experimental ablation at P0, hence a phasic drop in protein expression, may enable a feed-forward mechanism.

Notably, immunostaining for P27 ^Kip1^ and GLUL in Müller Glia is reduced both by overexpression^23^ and downregulation of *Lhx2* in the neonatal retina^9^, suggesting a balanced amount of LHX2 protein may be necessary to mediate the gliogenic process and a graded transcriptional response of target genes. Transcriptional de-regulation of *P27* ^*Kip1*^ and *Glul* may ultimately result from threshold responses that override the feed-forward regulatory loop controlling *Lhx2* expression.

LHX2 regulatory role likely relies on remodeling of nucleosomes units, variably coupled with active enhancers marks, flanked by active promoter and non-coding regions within modules of coordinately accessible chromatin. As a result, *Lhx2* loss-of-function affects genes involved in the cell cycle checkpoints, DNA replication, retinal disease phenotypes and the *Notch* signaling pathway, consistent with previous findings^9,10^. LHX2 has been previously shown to act as a regulator of genes that control cortical neuronal subtype identity by interaction with members of the nucleosome and histone deacetylase remodeling complex, although the mechanistic basis behind its regulatory role had not been investigated^24^.

LHX2 affects both local and global chromatin accessibility by competition for nucleosome occupancy, whereby secondary regulation of chromatin remodeling factors and pioneer factors is likely to concur to the widespread decrease in accessibility, observed upon *Lhx2* loss-of-function.

Secondary regulation of candidate pioneer factors may be achieved transcriptionally, by *Lhx2*-coordinated variations in local accessibility, paired with epistatic variations in gene expression, as is the case of *Sox2*, and/or by steric interaction of predicted co-factors, both of which would presumably result in altered transcription factors recruitment and nucleosome remodeling.

Transcriptional dependence of candidate pioneer factors on LHX2 can be exemplified with the following cases, wherein variations in local accessibility at their closest cis-regulatory sites bound by LHX2 co-occur with variations in expression levels upon *Lhx2* loss-of-function and/or across development. These genes include *Hes5*, *Neurod4, Meis1*, *Rax, Dlx2*, and *Neurod1*. Additional transcription factors, whose encoding genes are transcriptionally targeted by LHX2, exhibit high predicted pioneer potential such as ATF1, ATOH7, CREB1, DEAF1, EGR1, ETS2, ETV5, HEY1, HEYL, MYCN, NR2F2, PROX1,REST, SP4, TEAD1, VAX2, VSX2, ZBTB33, and ZFX.

The E14 retinal transcriptome shows greater *Lhx2*-dependence than would be expected based on peaks association by proximity to the closest promoter region. This may partially reflect the preponderant distribution of LHX2-binding sites at intergenic regions, exerting distal regulation of target genes. On the other hand, the extent to which *Lhx2* loss-of-function impacts retinal gene expression in early RPCs, may depend on collateral transcriptional networks evoked by LHX2, whereby the observed variations in gene expression following E14 *Lhx2* loss-of-function would be amplified by *Lhx2*-dependent transcription factors.

Among the many transcription factors predicted to co-occur with LHX2 ChIP-Seq peaks, some had predicted pioneer potential for which we observed variations in footprints counts upon *Lhx2* loss-of-function at E14. Affinity might be affected at both stages for many of the predicted interactors, as shown in their meta-profiles distribution and mean scores from motif centers. Moreover, nucleosomes depletion was observed at motif center of NF-I, KLF4/9, ASCL1, and HES5, while variations in nucleosomes coverage were detected upon *Lhx2* loss-of-function, suggesting LHX2 may be necessary to stabilize candidate pioneer co-factors prior to or during recruitment to target sites.

While several of the predicted pioneer transcription factors affected by LHX2, such as HES5, SOX8/9, and NFIA, play important roles in the control of retinal gliogenesis^17–20^, a process that is dependent on LHX2^10^, *Ascl1* plays an essential role in conferring neurogenic competence to late-stage RPCs^21,22^. Among additional LHX2 targets, *Pax6* and *Sox2* instead show broad temporal expression profiles^25,26^ and exert distinct functions across development^27–31^. Some of the known and candidate pioneer factors that exhibit developmentally regulated nucleosome-binding signatures, like NF-I, SOX2, KLF4, may preserve chromatin in a highly compacted state at embryonic states, while mediating chromatin unfolding later in development.

## Methods

### Experimental design

The experimental pipeline involves the generation and integration of high-throughput sequencing libraries from purified fractions of murine retinal progenitor cells (RPCs). Fluorescence-activated cell sorting (FACS) was adopted to isolate RPCs from mice expressing the RPC-specific *Chx10-Cre:eGFP* transgene^12^ from embryonic day (E)14 and post natal day (P)2 retina, representing early and late stages of RPC competence, respectively. Flow-sorted cells were profiled by RNA-Seq and ATAC-Seq, relying on direct in vitro transposition of sequencing adapters into native chromatin. ChIP-Seq was then performed on a select transcription factor candidate, identified in the preliminary screening of the ATAC-Seq-derived open chromatin regions. ChIP-Seq-binding sites for the candidate factor were ultimately integrated and compared with the age-matched RNA-Seq and ATAC-Seq profiles from control and knock-out retinas. For each experimental condition involving a point-source factor and broad regions (ChIP-Seq and ATAC-Seq, respectively) a minimum of 20 million uniquely mappable reads or ≥10 million uniquely mappable reads for each biological replicate were collected, according to the ENCODE’s guidelines. For point-source datasets, non-redundant mapped reads were retained for downstream analysis.

### Animals

CD-1 mice of either sex were euthanized at embryonic day 14 (E14) and post natal day 2 (P2) according to Johns Hopkins IACUC-approved protocols. Timed pregnant CD-1 mice were obtained from Charles River Laboratories. *Chx10-Cre:GFP* mice^12^ were purchased from the Jackson Laboratories. Retinas were freshly dissected, incubated in a suspension of papain and DNAse for 30 min at 37 °C, inactivated with bovine serum albumin, resuspended in equilibrated Earle’s balanced salt solution and subject to fluorescence-activated cell sorting (98–99% purity) where viability was assessed by propidium iodide exclusion. Cell fractions were collected on poly-d-lysine coated slides, fixed in 4% paraformaldehyde for 10 min, permeabilized in TritonX-100 and stained for CHX10 (Cat.# X1179P, Exalpha), GFP (Cat.# 600-101-215, Rockland), mKi67 (Cat.# RM-9106-S1,Thermo Scientific), or CCND1 (Cat.# SC-450, Santa Cruz). The brightest fraction, differing of fourfold mean intensity for GFP relative to the dim fraction, was always retained for subsequent processing and hereafter referred to as GFP-positive, RPC-enriched fraction. *Lhx2* conditional embryonic knockouts were obtained by crossing *Chx10-Cre:GFP* with *Lhx2*^*lox/lox*^ mice, and harvesting at E14^32^. Post natal *Lhx2* knockouts were generated by electroporation of pCAG-*Cre-GFP* construct into P0.5 wild-type CD-1 animals or *Lhx2*^*lox/lox*^ animals. Retinas were harvested at P2, dissociated, and GFP-positive electroporated cells were isolated by FACS. Overall electroporation efficiency was 2–3%.

### ATAC-Seq, RNA-Seq, and ChIP-Seq analysis

Chromatin derived from flow-sorted *Chx10-Cre-*GFP + ve and GFP-ve retinal fractions was processed as previously described^13^. Briefly, chromatin was extracted and processed for Tn5-mediated tagmentation and adapter incorporation, according to the Manufacturer’s protocol (Nextera DNA sample preparation kit, Illumina®) at 37 °C for 30 min. Reduced-cycle amplification was carried out in presence of compatibly indexed sequencing adapters. The quality of the libraries was assessed by fluorometric DNA incorporation-based assay (Thermo Fisher Scientific^TM^) and automated capillary electrophoresis (Agilent Technologies, Inc.) and up to four samples per lane were pooled and run as 50 bp paired ends on a HiSeq2500 Illumina sequencer.

RNA was processed with Qiagen RNAeasy Mini kit, subject to DNAse digestion, and samples with a minimum RNA integrity number (RIN) of seven were further processed for sequencing. Libraries were prepared using Illumina TruSeq RNA Sample kit (Illumina, San Diego, CA) following the manufacturer’s recommended procedure. Briefly, total RNA was denatured at 65 °C for 5 min, cooled on ice, purified and incubated at 80 °C for 2 min. The eluted mRNA was fragmented at 94 °C for 8 min and converted to double stranded cDNA, end repaired, A-tailed, and ligated with indexed adaptors and run on a MiSeq Illumina sequencer. The quality of the libraries was assessed by fluorimetric RNA incorporation-based assay (Thermo Fisher Scientific^TM^) and automated capillary electrophoresis (Agilent Technologies, Inc).

ChIP was performed as described previously^33^. Whole dissected retinas were dissociated in a collagenase I suspension, cross-linked in 1% formaldehyde for 15 min, and quenched in 125 mM glycine. The extracted nuclei were sheared to produce 100–500 bp fragments by means of probe sonication. Chromatin was immunoprecipitated with anti-Lhx2 (Cat.# SC-19344, Santa Cruz Biotechnology), rabbit anti-H3K27Ac (Cat.# ab4729, Abcam), or the corresponding isotype controls (Abcam); retained on agarose beads (Invitrogen), washed and purified by organic extraction. Amplicons corresponding to the targeted transcription factors and other candidate cis-regulatory regions that exhibit LHX2-binding sites and syntenic unbound regions had been verified by SYBR qRT-PCR (Agilent Technologies) as we previously reported^10^. Amplicons exhibiting enrichment of anti-LHX2 fractions over isotype control include *Six6*, *Fgf15, Rax, Vsx2, Dct, Car2, NeuroD1, Neurod4*, and *Neurog2*. Libraries were processed according to the manufacturer’s protocol (TruSeq Nano DNA Library Prep Kit). The quality of the libraries was assessed by fluorometric DNA incorporation-based assay (Thermo Fisher) and automated capillary electrophoresis (Agilent Technologies) and libraries (100–150 bp single read, paired ends) were run on a HiSeq2500 Illumina sequencer.

### Peak calling and retinal gene ontology analysis

RNA-Seq reads were aligned to the mouse transcriptome (mm9 UCSC build) using Tophat2^34^, and differentially expressed genes (DEGs) were identified by Cuffdiffs^35^, imposing a cutoff *q*-val = 0.05 for pairwise comparison.

Bowtie2 was used for ChIP-Seq and ATAC-Seq read alignment on the mouse genome (mm9)^36^. Uniquely mappable reads from ChIP-Seq were retained for peak calling by MACSs^37^ (band width = 300, mfold = 5, 50, *d* = 200, max tags per position = 1, min false discovery rate (FDR) *q*-val cutoff = 1E-02, *λ* = 1000–10,000 bp unless indicated otherwise).

Open chromatin regions were identified in ATAC-Seq data using MACS2^37^. Correlations between open chromatin states identified by pairwise comparison of normalized read counts in P2 and E14 GFP-positive flow-sorted retinal progenitor cells was computed with Jaccard^38^. The Jaccard index was estimated as the number of peaks that overlap between two peak files, divided by the union of the two files.

Footprints and nucleosomes were identified as described previously^15,39,40^. Annotation was performed by proximity to the closest transcription start site.

High-confidence ChIP-Seq peaks were identified from at least two experimental replicates (Poisson *p*-val threshold = 0.0001, min FE = 4, FDR = 0.001, max tags per position = 1, normalization to input, or isotype control) and subject to comparison with ATAC-Seq peaks (hypergeometric test, ln *p*-val) where co-occurrence was defined by physical overlap, allowing a max distance of 20 bp from peak summits over 3000 bp for confocality. For direct comparison of ChIP-Seq and ATAC-Seq data, high-confidence peaks with the highest differential in accessibility between nucleosomal units and flanking nucleosomes-free, transposon-accessible regions, and a minimum distance of 300 bp were identified from two ATAC-Seq replicates.

Genes that showed enriched or specific expression in retinal progenitors were identified based on the RNA-Seq data generated in this study. Genes that showed cell-type enriched or specific expression in adult retina were extracted from other published studies using RNA-Seq, microarray-based analysis of isolated cells, in situ hybridization or immunohistochemistry of intact retina. Association of ChIP-Seq peaks with genes expressed in specific retinal cell types was evaluated by Fisher’s exact test and corrected for multiple hypothesis testing (Bonferroni–Hochberg).

### Motif enrichment analysis

Hierarchical clustering of probabilistically assigned motifs (Jaspar 2016 non-redundant core vertebrates)^41^ was done with the following parameters (linkage = average; similarity threshold cor = 0.6, ncor = 0.4, rl = 5, where ncor is Pearson’s correlation (cor) by relative alignment length (rl) divided by the overall alignment length. (*e*-val = *p*-val × enriched oligos)^42,43^.

For ChIP-Seq analysis, binomial probability analysis of regulatory transcription factor motifs was calculated in the overall set of high-confidence peaks (minimum of two normalized replicates). The regions size was empirically determined and motifs were found by cumulative binomial distribution of known position weight matrices assuming a random representation of decamers. Motif finding was performed on the repeats masked mm9 murine genome, optimized for the top enriched 20 putative motifs, randomized and repeated twice to estimate FDR^44^.

### Footprint analysis

FIMO^45^ was used to scan the genome for candidate binding sites of different transcription factors. We then used BPAC to identify the actual binding sites among these candidate sites^40^. The number of active binding sites was analyzed at E14 and P2. Genome-wide changes in footprint counts and nucleosome occupancy for individual transcription factor motifs were estimated for all candidate binding sites at E14 and P2. Footprints scores were calculated for LHX2, KLF9/13, NF-I (NFIA/B/X), and SOX2. *T*-test on mean footprints scores distributed 200 bp from motif centers were calculated for the paired control and *Lhx2* cKO conditions.

### Statistical analysis

Co-occurrence statistics for point-source and broad regions of interest was computed by hypergeometric test with a default minimum overlap of 1 bp, unless otherwise specified. Coverage was adopted as reproducibility metrics for ChIP-Seq and ATAC-Seq experimental replicates (fraction of reads/10 Million non-redundant uniquely mappable reads) and FPKM for RNA-Seq, where correlation was reported by Pearson’s or Spearman’s coefficient. Binomial probability analysis of regulatory transcription factor motifs was applied genome-wide to identify enriched position weight matrices (PWMs) and clustered by average linkage. Fisher’s exact test was adopted to compute gene ontology enrichment and corrected for multiple hypothesis (Bonferroni–Hochberg) and RNA-Seq-derived gene sets from flow-sorted retinal cell fractions and control vs. experimental conditions (*Lhx2* cKO) with *q*-val (FDR) < 0.05 were retained for downstream analysis, unless otherwise specified. Two-tailed *t*-test was adopted to compare the average footprints counts for candidate pioneer factors in control and *Lhx2* knock-out conditions.

### Reporting Summary

Further information on experimental design is available in the Nature Research Reporting Summary linked to this article.

## Supplementary information

Reporting Summary

Supplementary Information

Description of additional supplementary items

Supplementary Data 1

Supplementary Data 2

Supplementary Data 3

Supplementary Data 4

## Data Availability

Auxiliary data, files, and tables have been deposited in the GEO repository, Accession Number GSE99818. Metadata descriptions for the GEO repository are available in Supplementary Data 4. All data needed to evaluate the conclusions in the paper are present in the paper and/or the Supplementary Materials. Additional data are available from the authors upon request.
