# Supplementary material for: Epigenomic profiling of retinal progenitors reveals LHX2 is required for developmental regulation of open chromatin
Source: Commun Biol. 2019 Apr 25;2:142. doi: 10.1038/s42003-019-0375-9 (PMC6484012; doi:10.1038/s42003-019-0375-9)
Supplement: Supplementary file 1 — Reporting Summary [file 42003_2019_375_MOESM1_ESM.pdf]

natureresearch

Corresponding author(s): Cristina Zibetti

Reporting Summary

Nature Research wishes to improve the reproducibility of the work that we publish. This form provides structure for consistency and transparency in reporting. For further information on Nature Research policies, see Authors & Referees and the Editorial Policy Checklist.

Statistical parameters

When statistical analyses are reported, confirm that the following items are present in the relevant location (e.g. figure legend, table legend, main text, or Methods section).

n/a	Confirmed	
☐	☒	The exact sample size (*n*) for each experimental group/condition, given as a discrete number and unit of measurement
☐	☒	An indication of whether measurements were taken from distinct samples or whether the same sample was measured repeatedly
☐	☒	The statistical test(s) used AND whether they are one- or two-sided<br>*Only common tests should be described solely by name; describe more complex techniques in the Methods section.*
☐	☒	A description of all covariates tested
☐	☒	A description of any assumptions or corrections, such as tests of normality and adjustment for multiple comparisons
☐	☒	A full description of the statistics including central tendency (e.g. means) or other basic estimates (e.g. regression coefficient) AND variation (e.g. standard deviation) or associated estimates of uncertainty (e.g. confidence intervals)
☐	☒	For null hypothesis testing, the test statistic (e.g. *F*, *t*, *r*) with confidence intervals, effect sizes, degrees of freedom and *P* value noted<br>*Give P values as exact values whenever suitable.*
☒	☐	For Bayesian analysis, information on the choice of priors and Markov chain Monte Carlo settings
☐	☒	For hierarchical and complex designs, identification of the appropriate level for tests and full reporting of outcomes
☐	☒	Estimates of effect sizes (e.g. Cohen's *d*, Pearson's *r*), indicating how they were calculated
☐	☒	Clearly defined error bars<br>*State explicitly what error bars represent (e.g. SD, SE, CI)*

Our web collection on statistics for biologists may be useful.

Software and code

Policy information about availability of computer code

Data collection	Tophat2 v2.1.1, Cuffdiff v2.2.1, Bowtie2 2.2.5, MACS 1.4.2, MACS2 2.1.0, GREAT v3, Homer v4.8.3, RSAT, Jaspar 2016, FIMO 4.12, DNASE2TF 1.0,BPAC 0.1
Data analysis	Please see software versions above.

For manuscripts utilizing custom algorithms or software that are central to the research but not yet described in published literature, software must be made available to editors/reviewers upon request. We strongly encourage code deposition in a community repository (e.g. GitHub). See the Nature Research guidelines for submitting code & software for further information.

Data

Policy information about availability of data

All manuscripts must include a data availability statement. This statement should provide the following information, where applicable:

- Accession codes, unique identifiers, or web links for publicly available datasets
- A list of figures that have associated raw data
- A description of any restrictions on data availability

Auxiliary data, SRA files and tables have been deposited in the GEO repository, Accession Number GSE99818 at https://www.ncbi.nlm.nih.gov/geo/query/acc.cgi?acc=GSE99818. All data needed to evaluate the conclusions in the paper are present in the paper, Supplementary Materials and Additional supplementary items.

All the figures and tables were derived from the data repository, except for Fig. S1 (flow sorting panels) and Supp.Table 4 (Manually curated list of retinal gene expression where the source of raw data is reported).

Field-specific reporting

Please select the best fit for your research. If you are not sure, read the appropriate sections before making your selection.

- [x] Life sciences
- [ ] Behavioural & social sciences
- [ ] Ecological, evolutionary & environmental sciences

For a reference copy of the document with all sections, see nature.com/authors/policies/ReportingSummary-flat.pdf

Life sciences study design

All studies must disclose on these points even when the disclosure is negative.

Sample size	For each experimental condition involving a point-source factor and broad regions (ChIP-Seq and ATAC-Seq, respectively) a minimum of 20 Millions uniquely mappable reads or ≥10 millions uniquely mappable reads for each biological replicate were collected, according to the ENCODE's guidelines. For point-source datasets, non-redundant mapped reads were retained for downstream analysis.
Data exclusions	The quality of the libraries was assessed by fluorometric DNA/RNA incorporation based assay (Thermo Fisher ScientificTM) and automated capillary electrophoresis (Agilent Technologies, Inc.). Specifically, RNA was processed with Qiagen RNAeasy Mini kit, subject to DNAse digestion, and samples with a minimum RNA integrity number (RIN) of 7 were further processed for sequencing. Libraries were prepared using Illumina TruSeq RNA Sample kit (Illumina, San Diego, CA) following manufacturer's recommended procedure. For ATAC-Seq, direct in vitro transposition of sequencing adapters into native chromatin was verified as first described in (Buenrostro et al. Nature Methods 10, 1213–1218 (2013)) to assess periodic patterns of approximately 200bp internucleosomal distances to ensure highly reproducible patterns of Tn5 tagmentation and coverage for open chromatin regions. The libraries were assessed according to the Manufacturer's protocol (Nextera DNA sample preparation kit, Illumina®). For ChIP-Seq, Libraries were processed according to the manufacturer's protocol (TruSeq Nano DNA Library Prep Kit). The quality of the libraries was assessed by fluorometric DNA incorporation based assay (Thermo-Fisher) and automated capillary electrophoresis (Agilent Technologies). Furthermore, an aliquot was taken from ChIP samples and verified for ChIP-qPCR enrichment versus isotype control and for percentage of input recovery of known targets. Amplicons corresponding to the targeted TFs and other candidate cis-regulatory regions that exhibit Lhx2-binding sites and syntenic unbound regions had been verified by SYBR qRT-PCR (Agilent Technologies). Examples of amplicons exhibiting statistically significant enrichment of anti-Lhx2 fractions over isotype control include Six6, Fgf15, Rax, Vsx2, Dct, Car2, NeuroD1, Neurod4 and Neurog2. Any library that did not pass quality standards was excluded from sequencing.
Replication	See criteria for data inclusion/exclusion above
Randomization	n/a. See criteria for data inclusion/exclusion above.
Blinding	N/A. Peak calling algorithms require to specify experimental condition and control samples so that normalization and enrichment of a given signal can be computed over stochastic distribution of the reads. Experimental conditions were always compared to control conditions for peak calling, differential accessibility of open chromatin regions or differentially expressed genes. IP fraction compared to isotype control, input samples (ChIP-Seq). GFP+ retinal progenitor cells fractions to GFP- post-mitotic fractions (RNA-Seq and ATAC-Seq).

Reporting for specific materials, systems and methods

Materials & experimental systems

n/a	Involved in the study	
☒	☐	Unique biological materials
☐	☒	Antibodies
☒	☐	Eukaryotic cell lines
☒	☐	Palaeontology
☐	☒	Animals and other organisms
☒	☐	Human research participants

Methods

n/a	Involved in the study	
☐	☒	ChIP-seq
☐	☒	Flow cytometry
☒	☐	MRI-based neuroimaging

Antibodies

Antibodies used	Chx10 (Cat.# X1179P, Exalpha), GFP (Cat.# 600-101-215, Rockland), Ki67 (Cat.# RM-9106-S1,Thermo Scientific) or Ccnd1 (Cat.# SC-450, Santa Cruz; anti-Lhx2 (Cat.# SC-19344, Santa Cruz Biotechnology), rabbit anti-H3K27Ac (Cat.# ab4729, Abcam)
Validation	For Ki67 (Cat.# RM-9106-S1,Thermo Scientific) see Wang P. et al. (Nat Med. 2015 Apr; 21(4): 383–388). Chx10 (Cat.# X1179P, Exalpha), GFP (Cat.# 600-101-215, Rockland), Ccnd1 (Cat.# SC-450); anti-Lhx2 (Cat.# SC-19344, Santa Cruz Biotechnology), rabbit

anti-H3K27Ac (Cat.# ab4729, Abcam) had been previously validated by retinal immunostaining or ChIP-qPCR (J Neurosci. 2016 Feb 24; 36(8): 2391–2405).

Animals and other organisms

Policy information about studies involving animals; ARRIVE guidelines recommended for reporting animal research

Laboratory animals	CD1 mice of either sex were euthanized at embryonic day 14 (E14) and postnatal day 2 (P2) according to Johns Hopkins IACUC-approved protocols. Timed pregnant CD-1 mice were obtained from Charles River Laboratories. Chx10-Cre:GFP mice were purchased from the Jackson Laboratories.
Wild animals	The study did not involve wild animals.
Field-collected samples	The study did not involve samples collected from the field.

ChIP-seq

Data deposition

☒ Confirm that both raw and final processed data have been deposited in a public database such as GEO.

☒ Confirm that you have deposited or provided access to graph files (e.g. BED files) for the called peaks.

Data access links<br>*May remain private before publication.*	https://www.ncbi.nlm.nih.gov/geo/query/acc.cgi?acc=GSE99818
Files in database submission	e14wt_703_AGGCAGAA_L002_R1_001.fastq.gz, e14wt_703_AGGCAGAA_L002_R2_001.fastq.gz, e14wt_705_GGACTCCT_L002_R1_001.fastq.gz, e14wt_705_GGACTCCT_L002_R2_002.fastq.gz, H3TG2BCXX_2_CGTACTAG_1.fastq.gz, H3TG2BCXX_2_CGTACTAG_2.fastq.gz, H3TG2BCXX_2_TAAGGCGA_1.fastq.gz, H3TG2BCXX_2_TAAGGCGA_2.fastq.gz, H2YTFBCXX_2_TAAGGCGA_1.fastq.gz, H2YTFBCXX_2_TAAGGCGA_2.fastq.gz, H53N5BCXX_2_TAAGGCGA_1.fastq.gz, H53N5BCXX_2_TAAGGCGA_2.fastq.gz, H7MJ2BCXX_2_TAGGCATG_1.fastq.gz, H7MJ2BCXX_2_TAGGCATG_2.fastq.gz, H7MJ2BCXX_2_AGGCAGAA_1.fastq.gz, H7MJ2BCXX_2_AGGCAGAA_2.fastq.gz, H7MJ2BCXX_1_TAAGGCGA_1.fastq.gz, H7MJ2BCXX_1_TAAGGCGA_2.fastq.gz, H7MJ2BCXX_1_CGTACTAG_1.fastq.gz, H7MJ2BCXX_1_CGTACTAG_2.fastq.gz, HCCGGADXX_1_CGTACTAG_1.fastq.gz, HCCGGADXX_1_CGTACTAG_2.fastq.gz, HCCGGADXX_1_TAAGGCGA_1.fastq.gz, HCCGGADXX_1_TAAGGCGA_2.fastq.gz, HHL3NBCXX_1_GCCAAT_1.fastq.gz, HHL3NBCXX_1_GCCAAT_2.fastq.gz, HHL3NBCXX_2_ACAGTG_1.fastq.gz, HHL3NBCXX_2_ACAGTG_2.fastq.gz, H2GNWBCXX_1_GCCAAT_1.fastq.gz, H2GNWBCXX_1_GCCAAT_2.fastq.gz, H2GNWBCXX_1_CTTGTA_1.fastq.gz, H2GNWBCXX_1_CTTGTA_2.fastq.gz, H9C83ADXX_2_GCCAAT_1.fastq.gz, H9C83ADXX_2_GCCAAT_2.fastq.gz, HB7KFADXX_2_ACAGTG_1.fastq.gz, HB7KFADXX_2_ACAGTG_2.fastq.gz, HCKKMBCXX_2_ACAGTG_1.fastq.gz, HCKKMBCXX_2_ACAGTG_2.fastq.gz, HCKKMBCXX_2_GTGAAA_1.fastq.gz, HCKKMBCXX_2_GTGAAA_2.fastq.gz, HCKKMBCXX_1_GCCAAT_1.fastq.gz, HCKKMBCXX_1_GCCAAT_2.fastq.gz, HCKKMBCXX_1_CTTGTA_1.fastq.gz, HCKKMBCXX_1_CTTGTA_2.fastq.gz, C14BUACXX_7_ACTTGA_1.fastq.gz, C14BUACXX_7_ACTTGA_2.fastq.gz, C14BUACXX_8_TAGCTT_1.fastq.gz, C14BUACXX_8_TAGCTT_2.fastq.gz, HJGYCBCXX_1_GTGAAAC_1.fastq.gz, HJGYCBCXX_1_ACAGTGA_1.fastq.gz, HJGYCBCXX_2_GCCAATA_1.fastq.gz, HVGL2BCXX_2_CGATGTA_1.fastq.gz, HVGL2BCXX_2_CAGATCA_1.fastq.gz, HVGL2BCXX_2_GTGAAAC_1.fastq.gz, H0A2UDAXXs_2_1_merged.fastq.gz, H13FHADXX_1_CTTGTA_1.fastq.gz, H140CADXX_1_GCCAAT_1.fastq.gz, H140CADXX_1_ACAGTG_1.fastq.gz, inpp2_HAEFKADXX_2_GTGAAA_1.fastq.gz, H7W7TBCXX_2_TGACCA_1.fastq.gz, H7W7TBCXX_2_CCGTCC_1.fastq.gz, E14_Control_ATAC-Seq_OCR.xlsx, E14_Lhx2_cKO_ATAC-seq_OCR.xlsx, E14_GFP_Plus_ATAC-Seq_OCR.xlsx, P2_Control_ATAC-Seq_OCR.xlsx, P2_Lhx2_cKO_ATAC-Seq_OCR.xlsx, P2_GPF_Plus_ATAC_Seq_OCR.xlsx, ATAC-Seq_Diff_OCRs.xlsx, RNA-seq_E14P2_WTKO.xlsx, RNA-seq_E14P2_RPC.xlsx, E14_Lhx2_ChIP-seq_peaks.xls, E14_H3K27AC_ChIP-seq_peaks.xls, P2_Lhx2_ChIP-seq_peaks.xlsx, P2_H3K27AC_ChIP-seq_peaks.xlsx
Genome browser session<br>(e.g. UCSC)	https://genome.ucsc.edu/cgi-bin/hgTracks?hgS_doOtherUser=submit&hgS_otherUserName=samal&hgS_otherUserSessionName=mm9_Lhx2

Methodology

Replicates	For each experimental condition involving a point-source factor and broad regions (ChIP-Seq and ATAC-Seq, respectively) a minimum of 20 Millions uniquely mappable reads or ≥10 millions uniquely mappable reads for each biological replicate were collected, according to the ENCODE's guidelines. For point-source datasets, non-redundant mapped reads were retained for downstream analysis. For ChIP-Seq: 2-3 experimental replicates + 1 control. For ATAC-Seq and RNA-Seq 2 replicates per condition.
Sequencing depth	For each experimental condition involving a point-source factor and broad regions (ChIP-Seq and ATAC-Seq, respectively) a minimum of 20 Millions uniquely mappable reads or ≥10 millions uniquely mappable reads for each biological replicate were collected, according to the ENCODE's guidelines. For point-source datasets, non-redundant mapped reads were retained for downstream analysis. For ATAC-Seq, read length is 50bp, they are paired-end. For RNA-Seq, read length is 150bp, they are paired-end. For ChIP-Seq at E14, read length is 150bp, they are single-end. For ChIP-Seq at P2, read length is 100bp, they are single-end. For sequencing depth, percentage mappability and reproducibility please refer to Fig. S3D,E,F.
Antibodies	anti-Lhx2 c20 (Cat.# SC-19344X, TransCruz reagent, Santa Cruz Biotechnology): isotype control anti-goat (Cat.# ab37373, AbCAM); rabbit anti-H3K27Ac (Cat.# ab4729, Abcam); Rabbit IgG chip grade (Diagenode Cat #kch-504-250)

Peak calling parameters	Bowtie2 was used for ChIP-Seq reads alignment on the mouse genome (mm9). Uniquely mappable reads from ChIP-Seq were retained for peak calling by MACS2 (band width = 300, mfold =5, 50, d = 200, max tags per position =1, min FDR q-val cutoff = 1E-02, lambda = 1000-10000 bp). High-confidence ChIP-Seq peaks were identified from at least 2 experimental replicates (Poisson p-val threshold = 0.0001, min FE=4, FDR=0.001, max tags per position =1, normalization to input or isotype control).
Data quality	Number of peaks within FDR 5% and above 5 fold enrichment. E14 Lhx2 ChIP-seq: 4485 P2 Lhx2 ChIP-seq: 3960, E14 H3K27Ac ChIP-seq: 4515. P2 H3K27Ac ChIP-seq: 7812
Software	Bowtie2, MACS, Homer v4.8.3

Flow Cytometry

Plots

Confirm that:

☒ The axis labels state the marker and fluorochrome used (e.g. CD4-FITC).

☒ The axis scales are clearly visible. Include numbers along axes only for bottom left plot of group (a 'group' is an analysis of identical markers).

☒ All plots are contour plots with outliers or pseudocolor plots.

☒ A numerical value for number of cells or percentage (with statistics) is provided.

Methodology

Sample preparation	CD1 mice of either sex were euthanized at embryonic day 14 (E14) and postnatal day 2 (P2) according to Johns Hopkins IACUC-approved protocols. Timed pregnant CD-1 mice were obtained from Charles River Laboratories. Chx10-Cre:GFP mice (12) were purchased from the Jackson Laboratories. Retinas were freshly dissected, incubated in a suspension of papain and DNAse for 30 min at 37 C, inactivated with bovine serum albumin, resuspended in equilibrated Earle's balanced salt solution and subject to fluorescence activated cell sorting (98-99% purity) where viability was assessed by propidium iodide exclusion. Cell fractions were collected on poly-D-lysine coated slides, fixed in 4% paraformaldehyde for 10 min, permeabilized in TritonX-100 and stained for Chx10 (Cat.# X1179P, Exalpha), GFP (Cat.# 600-101-215, Rockland), Ki67 (Cat.# RM-9106-S1,Thermo Scientific) or Ccnd1 (Cat.# SC-450, Santa Cruz). The brightest fraction, differing of 4-fold mean intensity for GFP relative to the dim fraction, was always retained for subsequent processing and hereafter referred to as GFP-positive, RPC-enriched fraction. Lhx2 conditional embryonic knockouts were obtained by crossing Chx10-Cre:GFP with Lhx2lox/lox mice, and harvesting at E14 (30). Postnatal Lhx2 knockouts were generated by electroporation of pCAG-Cre-GFP construct into P0.5 wild type CD1 animals or Lhx2lox/lox animals. Retinas were harvested at P2, dissociated, and GFP-positive electroporated cells were isolated by FACS. Overall electroporation efficiency was 2-3%.
Instrument	DakoCytomation MoFlo (3 laser, 6 color) for cell sorting
Software	MoFlo v4.3.02 Build 2451
Cell population abundance	Please refer to the Supplemental Figure 1. FACS analysis of dissociated P2 wild type retinas stained with Chx10 antibody reveals that 45% of cells at this age are immunopositive. Percentage of GFP positive cell fractions from Chx10-Cre:eGFP are reported at each developmental stage in Fig.S1.
Gating strategy	E14 and P2 Chx10-Cre:eGFP retinas were dissociated, gated by GFP mean intensity and the brightest flow-sorted fraction retained for immunostaining with Chx10 and GFP. Chx10-Cre:eGFP dissociated retinas were gated based on relative GFP+ intensity. Dissociated cells were first empirically gated based on clusters of GFP+ intensity emerging before the saturation of the signal at the inflection points of the histogram of events. The GFP-positive, GFP-negative and the interposed, dim fractions were collected. The brightest fraction had higher size and lower granularity (FSC Lin/ SSC Lin) compared to the dim and negative fractions. The presumptive RPC fraction differs for 4-fold GFP intensity from the dim fraction and represents 60% of the overall cell population. Cell viability was assessed by propidium iodide exclusion. Representative plots are from P2 retinas. The brightest GFP+ fraction was stained for Chx10, GFP and Ki67 or Ccnd1. The brightest fractions were hereafter referred to as GFP+, were always separated from the dim fraction and retained for subsequent applications. Control and Lhx2lox/lox retinas were electroporated at P0 with pCAG-Cre; GFP construct, collected after 48 hrs and subject to flow sorting. Viability was assessed by propidium iodide exclusion.

☒ Tick this box to confirm that a figure exemplifying the gating strategy is provided in the Supplementary Information.
