# Supplementary material for: Epigenomic profiling of retinal progenitors reveals LHX2 is required for developmental regulation of open chromatin
Source: Commun Biol. 2019 Apr 25;2:142. doi: 10.1038/s42003-019-0375-9 (PMC6484012; doi:10.1038/s42003-019-0375-9)
Supplement: Supplementary file 2 — Supplementary Information [file 42003_2019_375_MOESM2_ESM.pdf]

Supplementary Materials:

Supplementary Figures 1-10

Supplementary Tables: 1-12

Supplementary References

Additional Supplementary items:

Supplementary Data: 1-3

Data files and auxiliary files, data repository: GEO, accession number GSE99818

Supplementary Figure 1

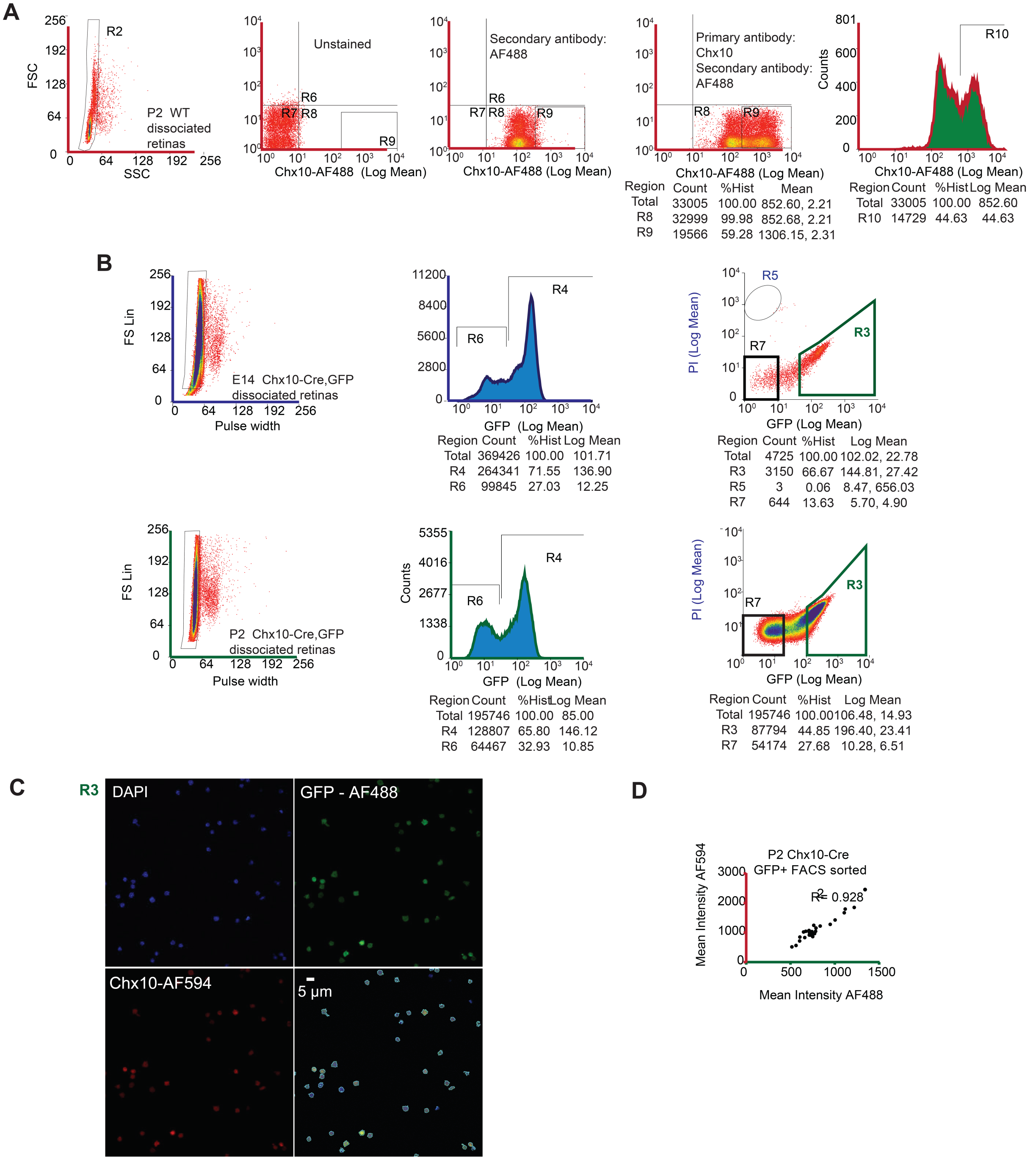

Supplementary Figure 1. (A-D) Retinal progenitor cells (RPCs) fractions from flow-sorted *Chx10-Cre:eGFP* retinas (A) FACS analysis of dissociated wildtype retinas at P2. Staining for VSX2 (*Chx10*) reveals that 45% of cells at this age are immuno-positive. **(B,C)** Dissociated

cells from *Chx10-Cre:eGFP* retinas at E14 and P2 were dissociated, gated by GFP mean intensity (B) and the brightest flow-sorted fraction was retained for immunostaining with VSX2 (*Chx10*) and GFP (C). **(D)** Correlation between the GFP reporter and P2 VSX2 (*Chx10*) immunofluorescence is shown.

Supplementary Figure 1

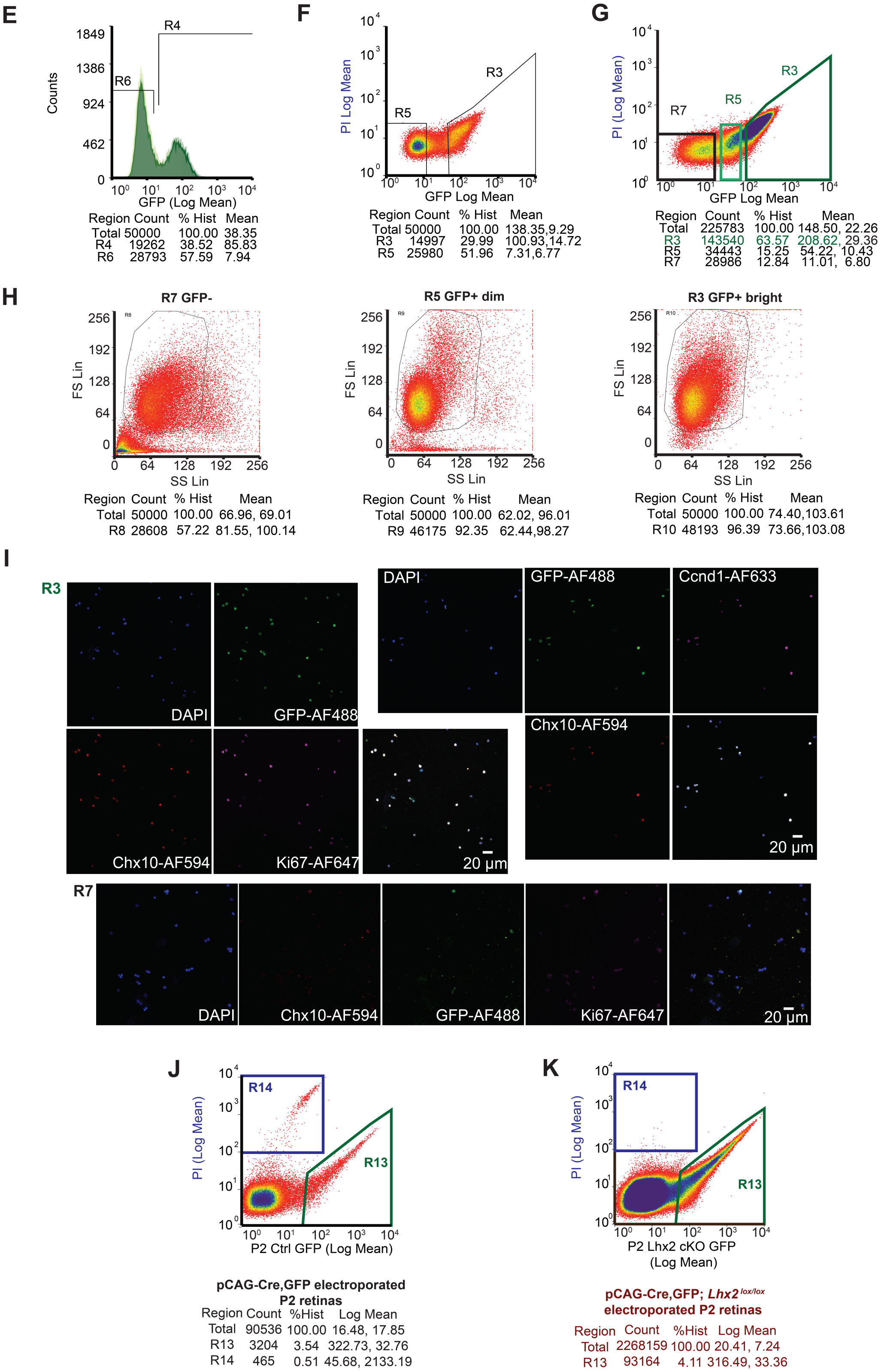

Supplementary Figure 1. (E,F) GFP intensity distribution for *Chx10-Cre:eGFP* dissociated retinal cells. Dissociated cells were first empirically gated based on clusters of GFP+ intensity

emerging before the saturation of the signal at the inflection points of the histogram of events. **(G)** The GFP-positive (R3), GFP-negative (R7) and the interposed, dim fractions (R5) were collected. **(H)** The brightest fraction had higher size and lower granularity (FSC Lin/ SSC Lin) compared to the dim and negative fractions. The presumptive RPC fraction shows a 4-fold higher GFP signal than the dim fraction and represents 60% of the overall cell population. Cell viability was assessed by propidium iodide exclusion. Representative plots are from P2 retinas. **(I)** The collected fractions were stained for VSX2 (*Chx10*), GFP and either mKi67 or CCND1. While both the bright and dim fractions are positive for VSX2 (*Chx10*) and GFP, only the brightest fraction (R3) stained for all the markers, it was hereafter referred to as GFP+, retained for gating, always separated from the dim fraction and utilized for subsequent applications. **(J,K)** Control and $Lhx2^{lox/lox}$ retinas were electroporated at P0 with *pCAG-Cre:GFP* construct, collected after 48 hrs and subject to flow sorting. Cell viability was assessed by propidium iodide exclusion.

Supplementary Figure 2

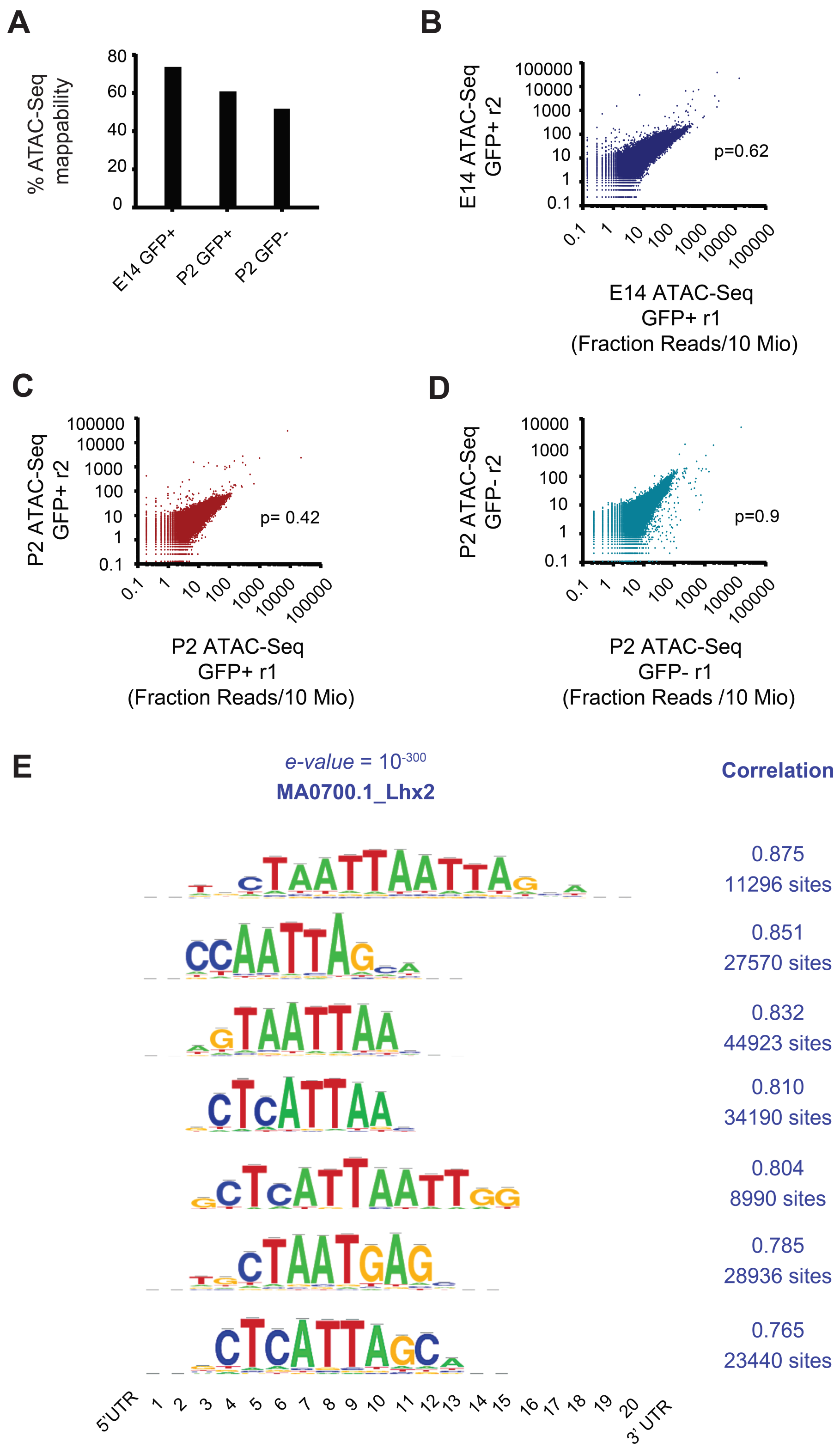

Supplementary Figure 2. LHX2 related motifs identified in open chromatin regions from early and late-stage RPCs. (A) Average mappability for ATAC-Seq replicates at E14 and P2

(**B-D**) Pearson's correlation of normalized read counts between replicates from E14 RPCs, P2 RPCs and P2 post-mitotic cells **(E)** Representative LHX2 related motif logos and enrichment statistics (MA0700.1, k-mer sig=300, e-value=1e-300). Motif instances were compared to the canonical LHX2 motif (Pearson's correlation coefficient) (*1*,*2*).

Supplementary Figure 3

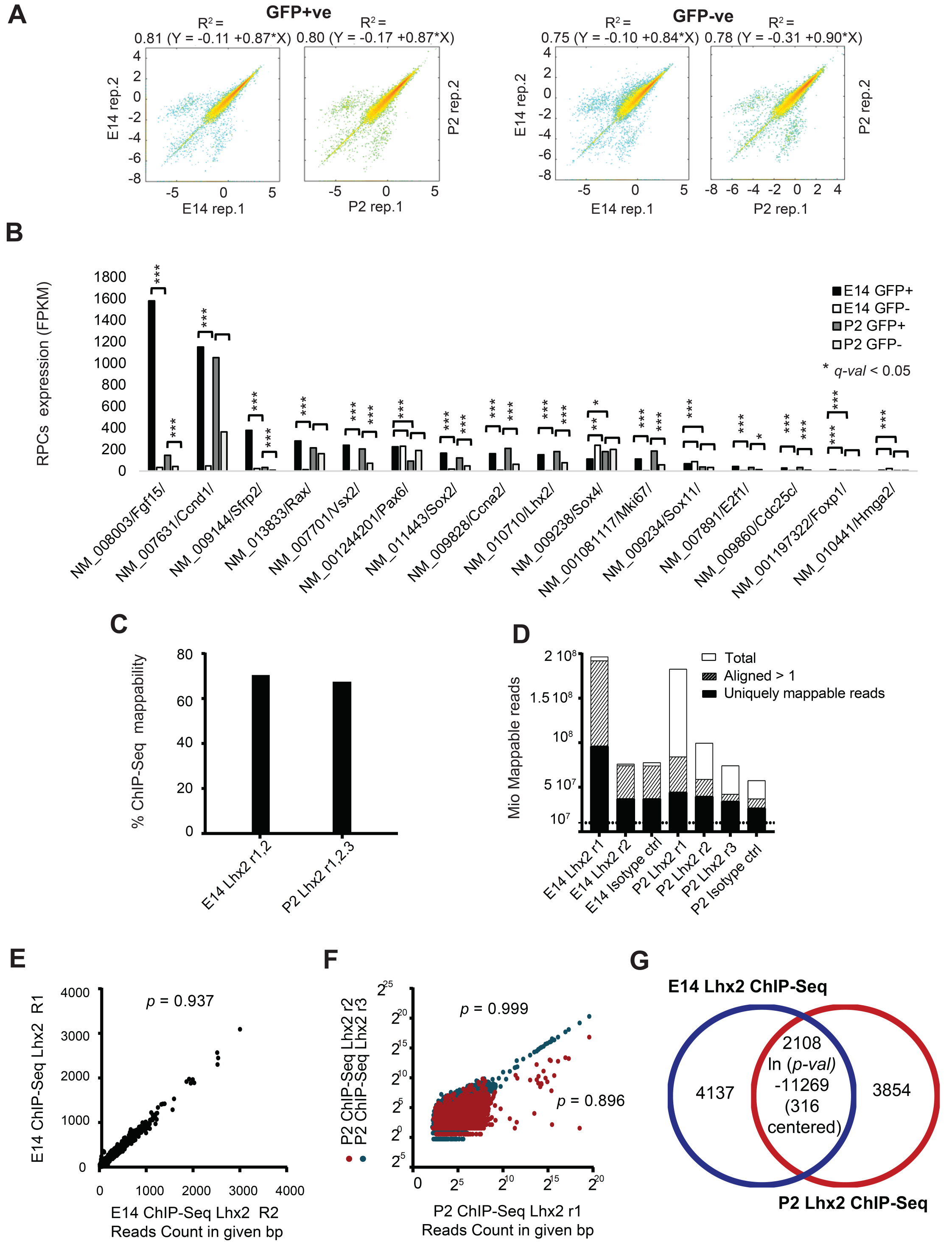

Supplementary Figure 3. Transcriptional profiling of flow sorted *Chx10*-GFP-positive RPCs and GFP-negative post-mitotic cells by RNA-Seq and integration with age-matched LHX2 ChIP-Seq. (A) Correlation between RNA-Seq replicates obtained from flow-sorted

Chx10-GFP cells at E14 and P2 **(B)** FPKM expression levels for *Lhx2* and other transcripts known to be expressed in RPCs. Asterisks indicate statistical significance (q-val < 0.05) for the comparison of transcripts from flow sorted GFP+ (R3) and GFP- (R5) fractions **(C,D)** LHX2 ChIP-Seq mappability for the E14 and P2 replicates (*3*). **(E,F)** Pearson's correlation of normalized reads counts for the LHX2 ChIP-Seq replicates at embryonic (E) and postnatal (F) stages. **(G)** High confidence LHX2 ChIP-Seq peaks at E14 and P2 were identified in at least two experimental replicates and retained for further analysis (R1,R2 for E14, r1,r2,r3 for P2). Peaks were called by MACS based on read counts averaged across replicates and normalized to age-matched isotype controls. Pairwise co-occurrence statistics for ChIP-Seq replicates is reported hereafter (Poisson p-val threshold = 0.0001, min FE=4, FDR=0.001, max tags per position =1) as detected in the experimental replicates at P2 [ln p-val (-36159,-36541,-52233,), ln Ratio Obs/Exp (4.16, 4.52,4.430), and E14 [ln p-val(-9271) ln Ratio Obs/exp (6.65)] (*4*). The Lhx2 known motif ranks first in 5/5 LHX2 ChIP-Seq replicates.

Supplementary Figure 4

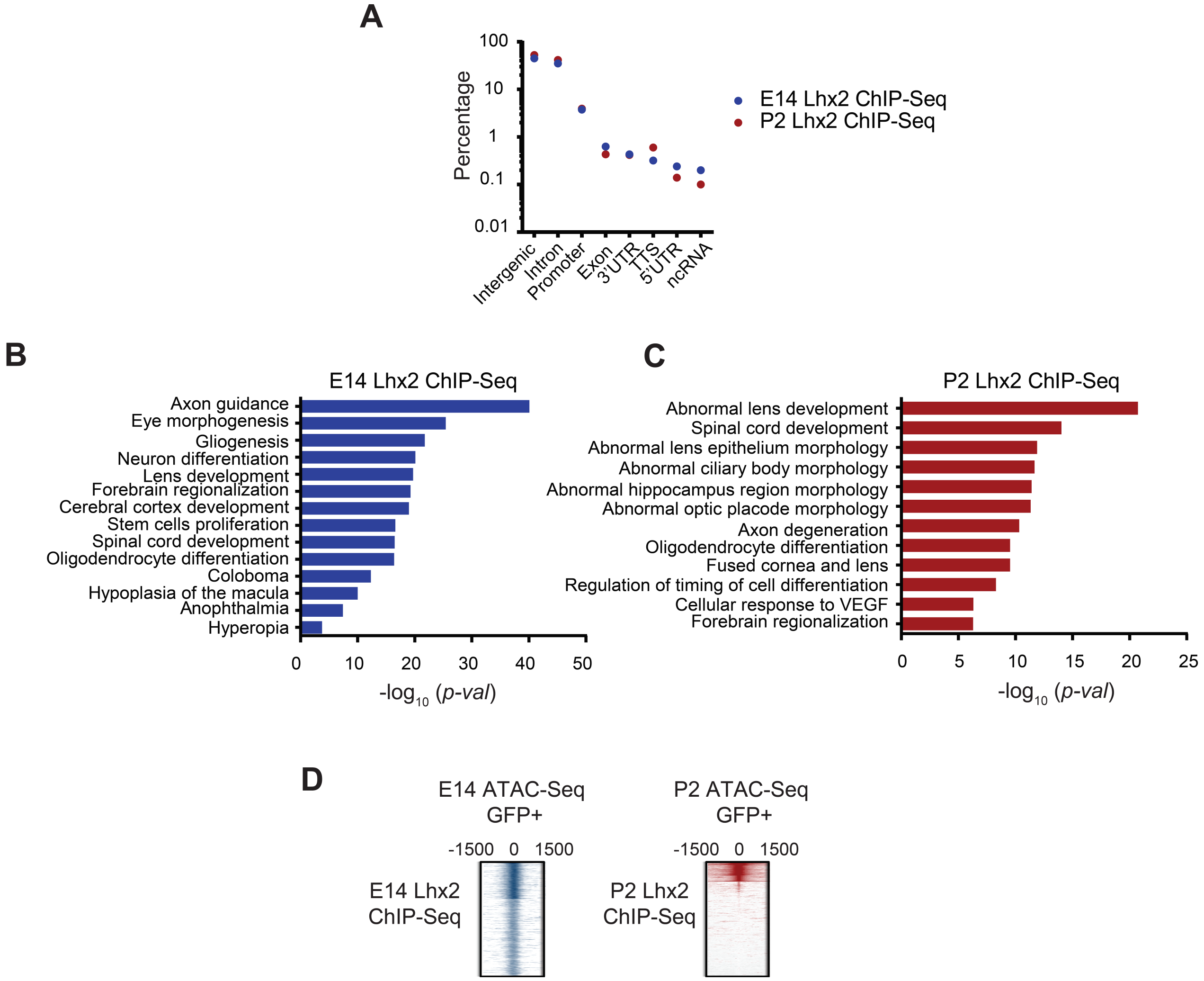

Supplementary Figure 4. Functional annotation of LHX2 ChIP-Seq peaks and integration with age-matched ATAC-Seq data. A. Prevalence of genomic regions overlapped with LHX2 ChIP-Seq peaks (*4*). (**B,C**) Functional enrichment analysis for LHX2 ChIP-Seq datasets at E14 and P2 by binomial distribution. **(D)** Heatmaps of raw reads from ATAC-Seq data relative to the center of the age-matched LHX2 ChIP-Seq peaks, 1.5 Kb from peak center. For local accessibility data, refer to Supplementary Table 7.

Supplementary Figure 5

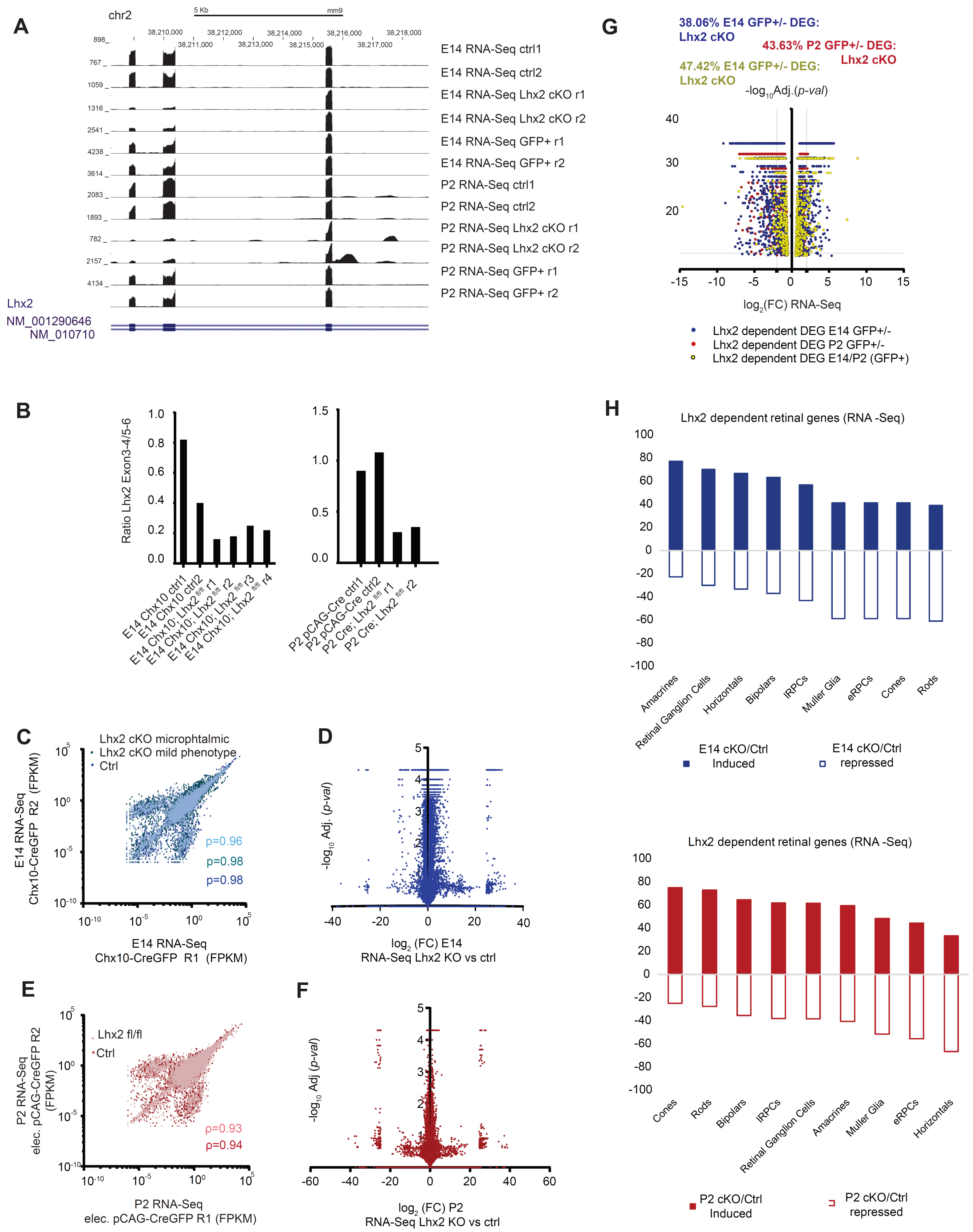

Supplementary Figure 5. Transcriptional profiling of flow-sorted RPCs from control and *Lhx2* cKO retinas and integration with LHX2 ChIP-Seq (A) UCSC custom tracks displaying

the RNA-Seq read distribution at the *Lhx2* locus in control and *Lhx2* cKO retinas at E14 and P2, indicating depletion of the reads at the excised region (intron 1-3). **(B)** Expression ratio between *Lhx2* exons 3-4 vs exons 5-6 demonstrates selective loss of expression of the *Lhx2* $^{lox/lox}$ allele in E14 and P2 *Lhx2* cKO retinas. (**C,D)** Distribution of differentially expressed genes (DEG) profiled by RNA-Seq from E14 *Lhx2* cKO replicates with moderate and severe microphthalmia (FPKM, Pearson's correlation coefficient) (*3*). **(E,F)** Distribution of DEG genes profiled by RNA-Seq from flow-sorted P2 *Lhx2* cKO replicates (FPKM, Spearman's correlation). DEG genes (FDR < 0.05) identified from the *Lhx2* cKO at E14 and P2 were utilized for downstream analysis and ChIP-Seq peak annotation (*3*). **(G)** Developmentally regulated genes displayed in the volcano plot were defined by pairwise comparison of the flow-sorted cell fractions at E14 and P2. Developmentally regulated, *Lhx2*-dependent genes (*Lhx2* cKO) exhibiting variations in at least one developmental time point are reported as percentage of all expressed genes. Non-redundant, unique Gene IDs counts are reported in Supplementary Table 5 and Supplementary Data 2.1-2.3. The relative proportion of DEGs that can be assigned by proximity to at least one LHX2 ChIP-Seq identified regulatory sequence is reported in percentage in Supplementary Table 4. The proportion of ChIP-Seq functional peaks associated to DEG, *Lhx2*-dependent genes is reported in Supplementary Table 9. (**H**) Percentage distribution of *Lhx2*-dependent retinal genes at E14 and P2 (Supplementary Data 2.4). Up-regulated and down-regulated DEGs are shown in filled and blank bars, respectively.

Supplementary Figure 6

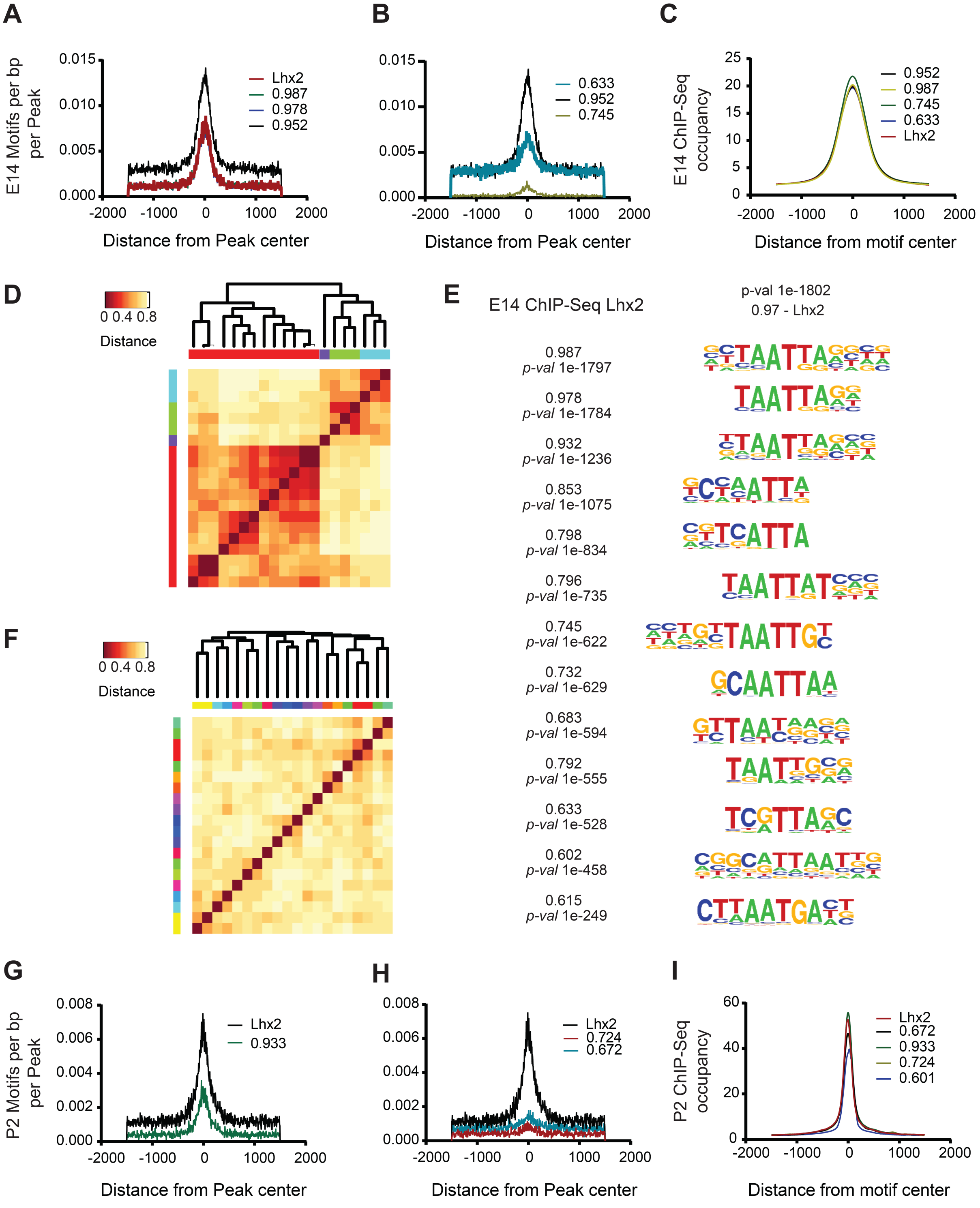

Supplementary Figure 6. Developmentally regulated occupancy of LHX2 motifs that diverge from the consensus. (A-B) LHX2 related motif frequency in ChIP-Seq peaks at E14.

Novel motifs are labeled by their Pearson's correlation coefficient to the known LHX2 motif (*4*). **(C)** LHX2 ChIP-Seq occupancy is reported for those motifs identified at E14 diverging from the LHX2 consensus (*4*). **(D)** Clustering of LHX2 related motifs identified at E14 (linkage=average; similarity threshold cor =0.6, ncor=0.4, w=5) (*2*) **(E)** Analysis of LHX2 regulatory motifs at E14. The most enriched cluster of E14 transcription factor binding motifs (D) comprises multiple instances of the LHX2 motif, each labelled by Pearson's correlation to the known LHX2 motif. Positional variations, statistical significance by binomial probability and assigned representative logos are also reported (*2*). (**F)** Clustering of permuted LHX2 motifs is displayed for comparison with those identified at E14 (D) (*2*). **(G,H)** LHX2 related motif frequency in ChIP-Seq peaks at P2. Pearson's correlation coefficients indicate similarity between the novel motifs identified by ChIP-Seq at P2 and the known LHX2 motif (*4*). (**I)** LHX2 ChIP-Seq occupancy is reported for those motifs identified at P2 diverging from the LHX2 consensus (*4*).

Supplementary Figure 7

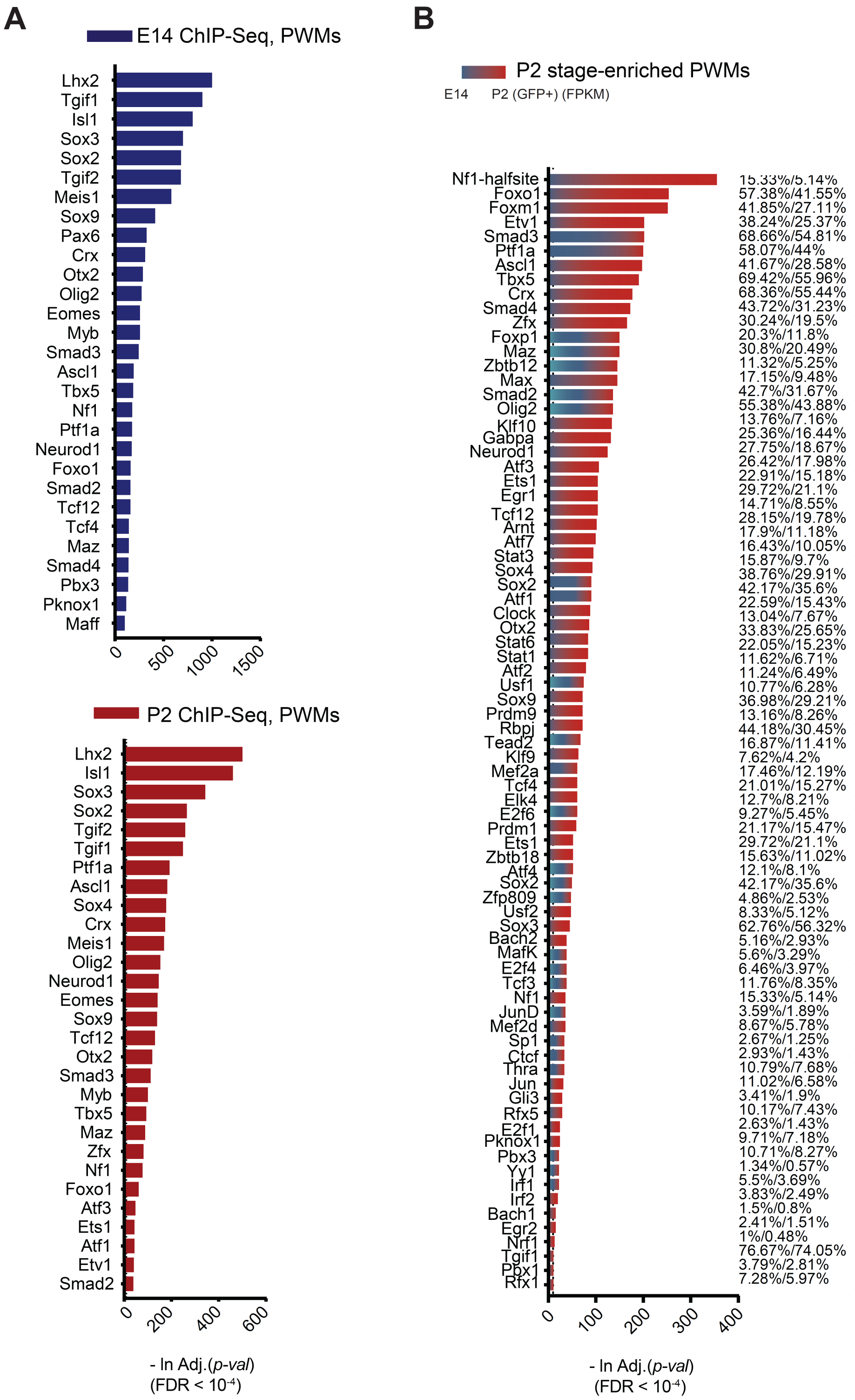

Supplementary Figure 7. Transcription factor motifs with known and putative pioneer function are among LHX2 co-occurrences at embryonic and post-natal stages. (A)

Enrichment of known transcription factor motifs for the E14 and P2 LHX2 ChIP-Seq datasets by binomial scoring of position weight matrices (*4*). (**B**) Comprehensive list of P2 preferential motifs is reported, with percentage of occurrence in P2 and E14 ChIP-Seq peaks, respectively (*4*). Relative expression levels of the corresponding transcription factor mRNA at E14 or P2 are indicated by the blue (E14) to red (P2) color gradient showing preferential expression at either time point.

Supplementary Figure 8

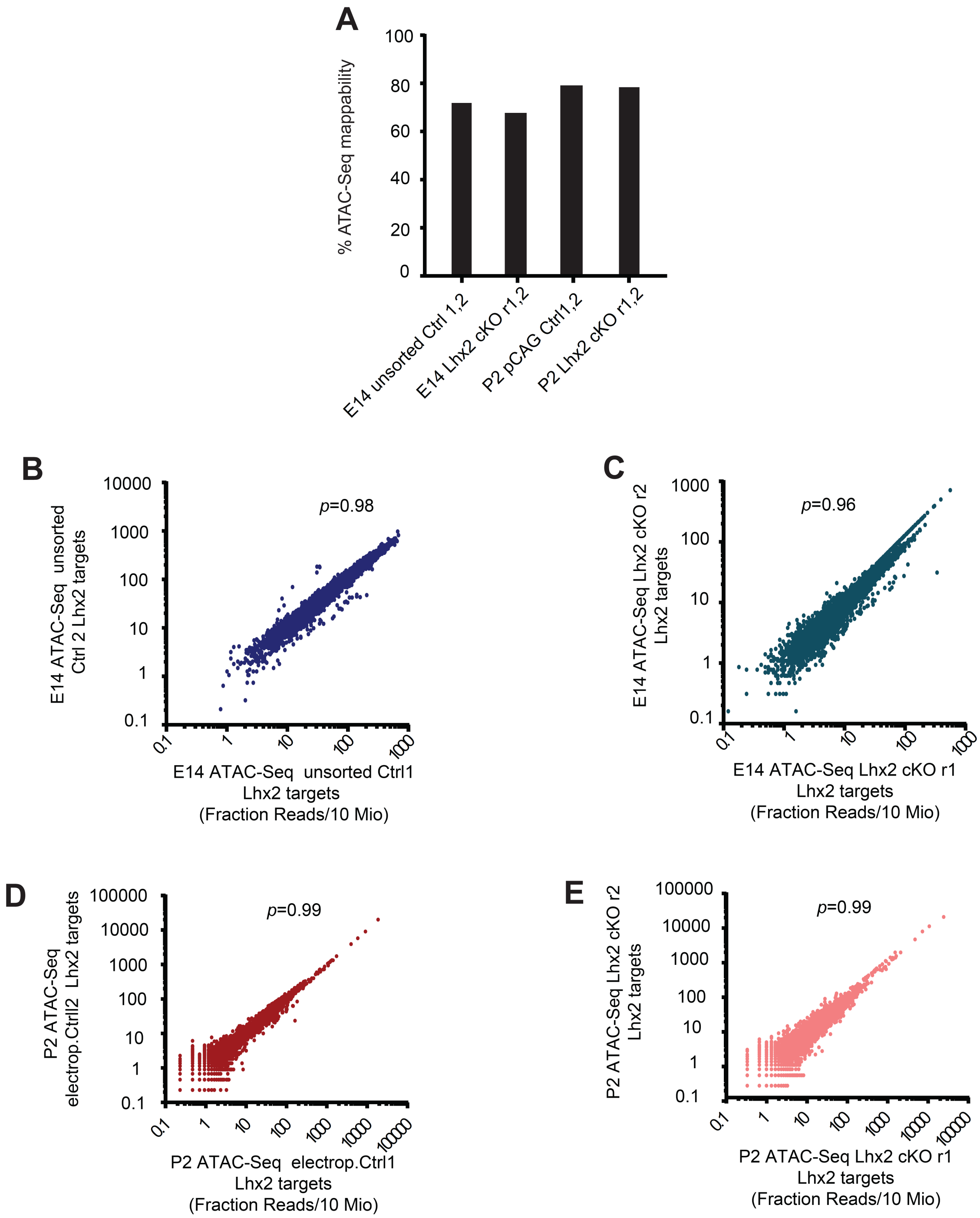

Supplementary Figure 8. LHX2 controls local and global chromatin accessibility in RPCs

(A-E) Average percentage mappability (A) and reproducibility for the ATAC-Seq replicates at

E14 (B,C) and P2 (D,E) in control conditions and *Lhx2* cKO (*4*). The read counts were normalized for the library size.

Supplementary Figure 9

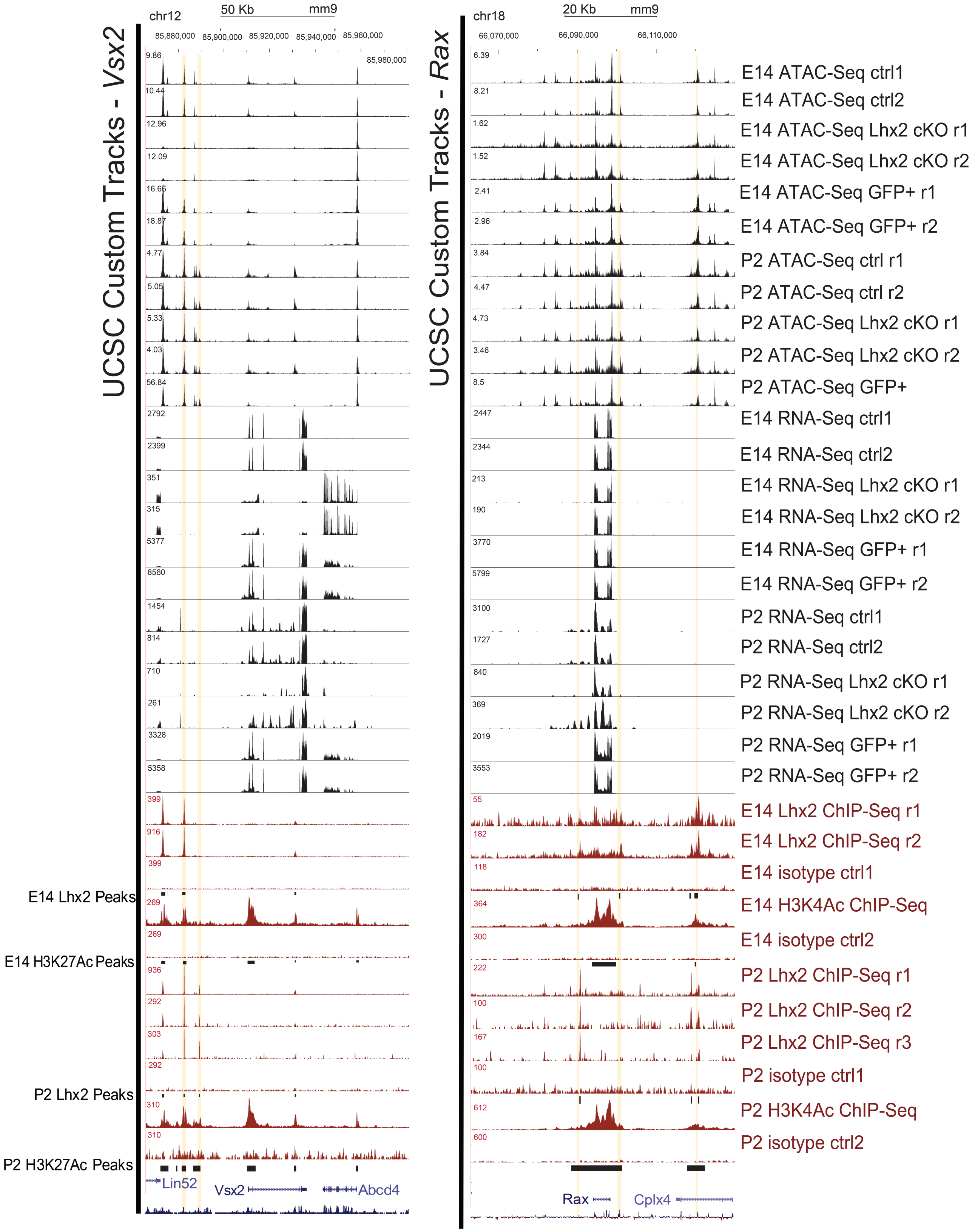

Supplementary Figure 9. ChIP-Seq, ATAC-Seq and RNA-Seq custom tracks configured for the LHX2 target genes *Vsx2* and *Rax*. UCSC custom tracks displaying the candidate cis-

regulatory loci for *Vsx2* and *Rax* in the murine genome, bound by LHX2 and validated by ChIP-qPCR (*5*,*6*). Age-matched ATAC-Seq and RNA-Seq expression levels from *Lhx2* control and cKO conditions are also displayed. The regions highlighted indicate candidate LHX2 cis-regulatory elements that exhibit LHX2 binding sites, variations in chromatin accessibility and in expression of the closest transcript in *Lhx2* cKO. Detailed paired variations in sequencing coverage at the *Vsx2* locus across datasets and footprinting analysis of altered transcriptional networks in Lhx2 cKO are also provided (Supplementary Data 3).

Additional candidate cis-regulatory sites bound by LHX2 were found in proximity to the TSS for *Neurod1, Neurod4, Neurog2, Olig2 and Ascl1.* LHX2 targets, first identified by ChIP-Seq, were subsequently cross-referenced against, by *in situ* hybridization in *Lhx2* cKO retinas at P0 (*Pdgfra-Cre*; *Lhx2* $^{\mathrm{lox/lox}}$) (*5*,*6*), by RNA-Seq (FPKM) in E14 (*Chx10-Cre;Lhx2* $^{lox/lox}$ retinas) and P2 retinas (*Lhx2* $^{lox/lox}$ electroporated with pCAG-*Cre-GFP* at P0 and isolated by FACS) (Supplementary Data 2) and by immunohistochemistry (*6*).

Supplementary Figure 10

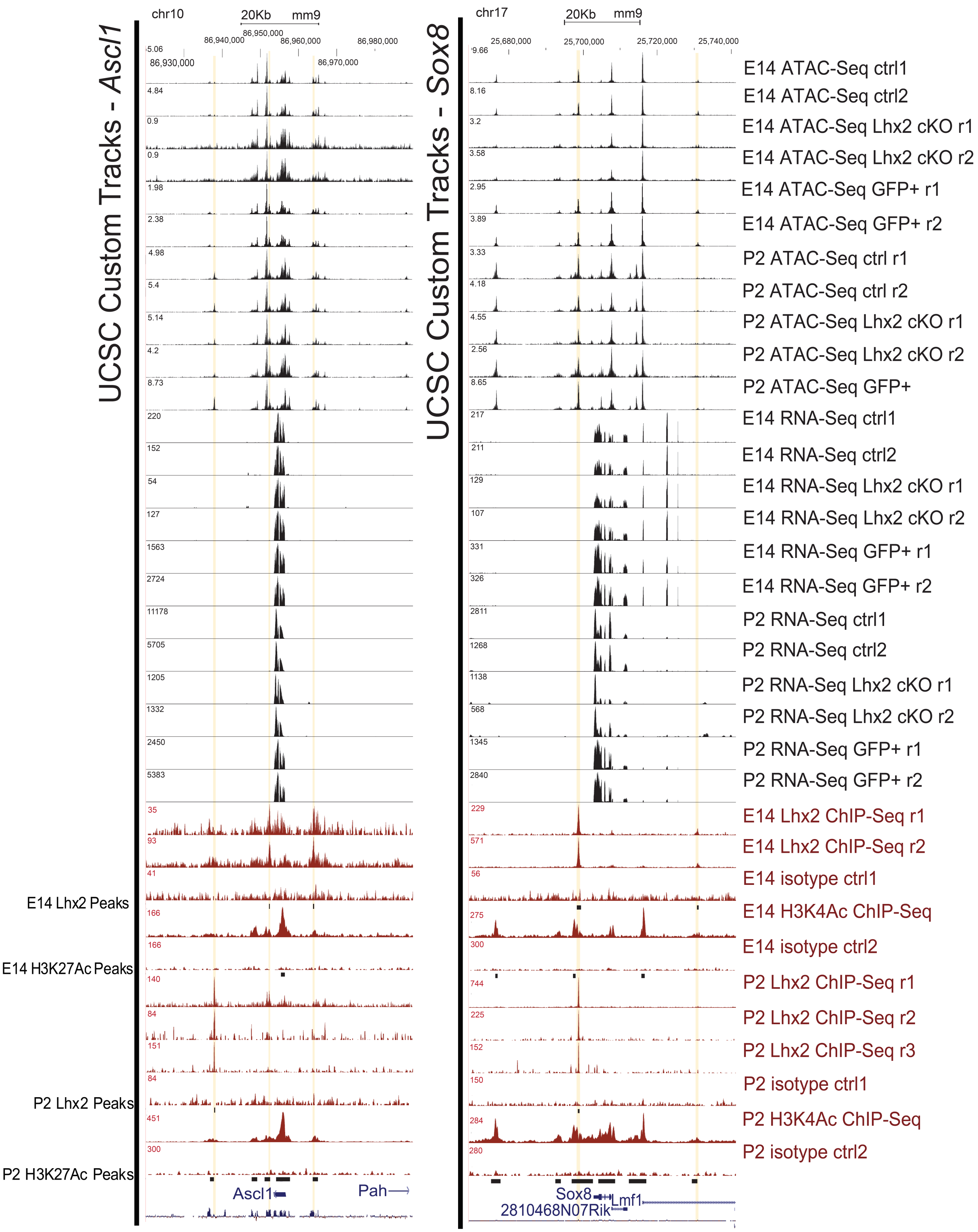

Supplementary Figure 10. ChIP-Seq, ATAC-Seq and RNA-Seq custom tracks configured for the LHX2 target genes *Ascl1* and *Sox8*. UCSC custom tracks displaying the candidate cis-

regulatory loci for *Ascl1* and *Sox8* in the murine genome, bound by LHX2 and validated by ChIP-qPCR (*6*). Age-matched ATAC-Seq and RNA-Seq expression levels from *Lhx2* control and cKO conditions are also displayed. The regions highlighted indicate candidate LHX2 cis-regulatory elements that exhibit LHX2 binding sites, variations in chromatin accessibility and in expression of the closest transcript in *Lhx2* cKO. Detailed paired variations in sequencing coverage at the *Sox8* locus across datasets and footprinting analysis of altered transcriptional networks in Lhx2 cKO are also provided (Supplementary Data 3).

Additional candidate cis-regulatory sites bound by LHX2 were found in proximity to the TSS for *Lhx2, Notch1, Dll1, Dll3, Hes5, Fgf15, Sfrp2, Otp, Lhx5 and Six6.* LHX2 targets, first identified by ChIP-Seq, were subsequently cross-referenced against, by *in situ* hybridization in *Lhx2* cKO retinas at P0 (*Pdgfra-Cre*; *Lhx2* $^{lox/lox}$) (*5,6*), by RNA-Seq (FPKM) in E14 (*Chx10-Cre;Lhx2* $^{lox/lox}$ retinas) and P2 retinas (*Lhx2* $^{lox/lox}$ electroporated with pCAG-*Cre-GFP* at P0 and isolated by FACS) (Supplementary Data 2) and with whole mount in situ (*7*). Additional LHX2 targets such as *Mki67ip, Cdkn1b (p27*^{Kip1}*), Glul, Rlbp1 and Sox9* were cross-referenced against by immunohistochemistry (*5*), where cells electroporated with pCAGIG-*Lhx2* at P0 showed downregulation of VSX2 and mKi67 by P2 (*6*). Retinal dissociates showing YFP and mKi67 co-labeling at P0 in *Pdgfra-Cre;R26-stop-YFP;Lhx2* $^{lox/lox}$ and *Pdgfra-Cre;R26-stop-YFP;Lhx2* $^{lox/lox}$ animals have also shown a reduction of YFP-labeled cells co-expressing mKi67 in *Pdgfra-Cre;R26-stop-YFP;Lhx2* $^{lox/lox}$ animals compared to controls at P0 and no proportional change in co-expression of mKi67 in YFP-ve cells in conditional knock-outs compared to controls *(5*). Electroporation of pCAG-*Cre* into *Lhx2* $^{lox/lox}$ mice at P0 also disrupts the development of Muller Glia as shown by reduction of glial markers P27^{Kip1},GLUL, RLBP1 and SOX9 in *Lhx2* $^{lox/lox}$ retinas compared to *Lhx2* $^{+/+}$ (*5*).

Supplementary Tables:

Known motifs in E14, P2 RPCs open chromatin regions										
	**p-val**	**ln p-val**	**q-val (Benj.)**	**% Target Seq**	**% Bckg Seq**	**ISH, FACS-microarray**	**E14 Chx10-GFP+ve (FPKM)**	**P2 Chx10-GFP+ve (FPKM)**	**log_2FC (FPKM) E14 RPCs vs P2 RPCs**	**q-val E14 vs P2 RPCs**
**Ctcf**	1E-5389	-12410	0	0.19	0.03	*	91.43	101.59	-0.15	0.763
**Lhx2**	1E-1449	-3337	0	0.35	0.21	*	152.71	183.84	-0.27	0.508
**Sox2**	1E-588	-1354	0	0.28	0.19	n/a	168.62	123.39	0.45	0.196
**Sox4**	1E-468	-1080	0	0.27	0.19	*	115.68	183.95	-0.67	0.014
**Neurod1**	1E-365	-841	0	0.28	0.21	*	60.64	218.59	-1.85	0.001
**Sp1**	1E-328	-758	0	0.15	0.10	n/a	29.88	39.14	-0.39	0.224
**Pax6**	1E-298	-688	0	0.04	0.02	*	226.36	95.15	1.25	0.001
**Tcf12**	1E-282	-649	0	0.33	0.26	*	27.45	42.97	-0.65	0.244
**E2f4**	1E-261	-602	0	0.17	0.12	n/a	57.13	45.66	0.32	0.381
**Sox9**	1E-258	-595	0	0.26	0.20	*	50.72	83.48	-0.72	0.006
**Otx2**	1E-243	-560	0	0.20	0.16	*	12.26	51.41	-2.07	0.056
**E2f6**	1E-231	-533	0	0.19	0.14	n/a	32.93	23.41	0.49	0.228
**Rfx1**	1E-223	-514	0	0.09	0.05	n/a	10.26	11.96	-0.22	0.635
**E2f1**	1E-188	-435	0	0.10	0.07	*	29.69	59.28	-1.00	0.010
**Elk4**	1E-160	-370	0	0.21	0.17	n/a	6.43	12.28	-0.93	0.001
**Elk1**	1E-143	-330	0	0.20	0.16	n/a	14.70	15.06	-0.03	0.958
**Tead2**	1E-136	-313	0	0.15	0.11	n/a	173.97	133.26	0.38	0.245
**E2f7**	1E-128	-295	0	0.05	0.03	n/a	26.28	22.05	0.25	0.532
**Egr1**	1E-119	-276	0	0.23	0.19	*	288.67	587.92	-1.03	0.005
**Meis1**	1E-109	-252	0	0.46	0.41	n/a	57.05	37.44	0.61	0.039
**Maz**	1E-99	-229	0	0.41	0.37	*	300.90	268.31	0.17	0.753
**Nrf1**	1E-85	-196	0	0.07	0.05	n/a	15.87	16.92	-0.09	0.916
**Myb**	1E-80	-186	0	0.43	0.40	n/a	96.55	70.08	0.46	0.510
**Usf1**	1E-72	-166	0	0.13	0.11	n/a	36.05	31.68	0.19	0.703
**Klf4**	1E-72	-166	0	0.14	0.11	n/a	11.12	7.30	0.61	0.098
**Tcf4**	1E-71	-165	0	0.12	0.10	*	19.15	67.98	-1.83	0.001
**NF1**	1E-66	-152	0	0.12	0.10	n/a	9.34	14.75	-0.66	0.024
**Smad2**	1E-63	-145	0	0.43	0.40	n/a	27.38	26.48	0.05	0.938
**Tgif2**	1E-61	-142	0	0.68	0.65	n/a	41.41	33.85	0.29	0.570
**Pbx3**	1E-61	-141	0	0.09	0.07	n/a	17.60	3.90	2.17	0.001
**JunD**	1E-56	-130	0	0.03	0.02	n/a	174.51	141.08	0.31	0.434
**Usf2**	1E-55	-127	0	0.09	0.07	n/a	28.81	34.02	-0.24	0.565
**Atf2**	1E-53	-124	0	0.09	0.08	n/a	10.11	8.92	0.18	0.884
**Smad4**	1E-53	-123	0	0.44	0.41	n/a	55.15	64.52	-0.23	0.580

**Pknox1**	1E-53	-123	0	0.08	0.07	n/a	14.81	19.31	-0.38	0.303
**Atf1**	1E-53	-122	0	0.17	0.15	n/a	21.63	19.86	0.12	0.840
**Pbx1**	1E-48	-112	0	0.03	0.02	n/a	5.27	6.34	-0.27	0.839
**Clock**	1E-48	-112	0	0.15	0.13	n/a	4.14	13.35	-1.69	0.001
**Gabpa**	1E-48	-111	0	0.27	0.25	n/a	28.97	34.13	-0.24	0.649
**Klf10**	1E-42	-97	0	0.18	0.16	n/a	10.48	18.94	-0.85	0.004
**Tgif1**	1E-40	-93	0	0.64	0.61	n/a	30.61	27.95	0.13	0.857
**Zbtb33**	1E-36	-85	0	0.02	0.01	n/a	6.16	9.94	-0.69	0.277
**Tbx20**	1E-34	-78	0	0.07	0.06	n/a	0.62	0.01	5.64	0.027
**Tcf3**	1E-32	-74	0	0.07	0.06	*	18.16	11.66	0.64	0.726
**Max**	1E-31	-72	0	0.17	0.15	n/a	21.72	23.55	-0.12	0.867
**Prdm1**	1E-30	-70	0	0.13	0.12	*	17.29	49.52	-1.52	0.001
**Gli3**	1E-28	-67	0	0.04	0.03	n/a	19.86	42.70	-1.10	0.001
**Foxp1**	1E-27	-64	0	0.10	0.09	*	17.01	1.81	3.23	0.001
**Foxo1**	1E-26	-62	0	0.43	0.41	n/a	4.34	6.67	-0.62	0.069
**Etv1**	1E-26	-61	0	0.37	0.35	*	1.78	5.42	-1.61	0.004
**THRa**	1E-26	-60	0	0.12	0.11	n/a	46.29	52.34	-0.18	0.698
**Atf4**	1E-23	-54	0	0.06	0.05	n/a	218.36	191.76	0.19	0.790
**Ptf1a**	1E-21	-49	0	0.60	0.58	*	13.94	4.76	1.55	0.001
**Zscan22**	1E-18	-44	0	0.03	0.02	n/a	2.98	3.43	-0.21	0.850
**Bach2**	1E-15	-35	0	0.05	0.04	n/a	5.01	6.16	-0.30	0.474
**YY1**	1E-11	-28	0	0.03	0.02	n/a	67.79	58.95	0.20	0.644
**Mef2d**	1E-10	-25	0	0.04	0.03	n/a	14.62	23.25	-0.67	0.025
**Bach1**	1E-10	-24	0	0.01	0.01	n/a	16.53	13.20	0.32	0.372
**Mef2a**	1.E-04	-10	0	0.08	0.08	n/a	10.18	9.46	0.11	0.880
**Foxm1**	1.E-03	-8	0.001	0.21	0.20	*	82.94	88.42	-0.09	0.877
**Zfx**	1	-1	0.247	0.34	0.33	n/a	3.92	6.00	-0.61	0.440
**Zbtb12**	1	0	1	0.11	0.11	n/a	73.24	38.54	0.93	0.001
**Tbx5**	1	0	1	0.64	0.65	n/a	2.62	3.41	-0.38	0.509

Supplementary Table 1. Transcription factors with known motifs that are enriched in open chromatin regions common to E14 and P2 RPCs

Known motifs for transcription factors expressed in early and late RPC from *Chx10-Cre:eGFP* retinas and overrepresented in regions of chromatin accessible at both time points (ATAC-Seq) are reported. Non-redundant IDs are listed, with the highest RNA-Seq reads counts (FPKM) for each transcription factor. Asterisk indicates evidence of expression in the retina (Supplementary Data 1).

Known motifs in E14 specific RPCs open chromatin regions										
	p-val	ln p-val	q-val (Benj.)	% of Target Seq	% of Bckg Seq	Expression by ISH,FACS-microarray	E14 Chx10-GFP+ve (FPKM)	P2 Chx10-GFP+ve (FPKM)	$\log_2$FC (FPKM) E14 RPCs vs P2 RPCs	q-val E14 vs P2 RPCs
**Tgif1**	1E-702	-1617	0	0.71	0.52	n/a	30.61	27.95	0.13	0.857
**Tgif2**	1E-622	-1433	0	0.72	0.54	n/a	41.41	33.85	0.29	0.570
**Meis1**	1E-608	-1401	0	0.46	0.29	n/a	57.05	37.44	0.61	0.039
**Tbx5**	1E-394	-909	0	0.58	0.44	n/a	2.62	3.41	-0.38	0.509
**Tead2**	1E-313	-722	0	0.17	0.09	n/a	173.97	133.26	0.38	0.245
**Ptf1a**	1E-219	-505	0	0.50	0.39	*	13.94	4.76	1.55	0.001
**Rfx2**	1E-196	-452	0	0.05	0.02	n/a	16.23	10.85	0.58	0.141
**Isl1**	1E-186	-429	0	0.55	0.45	n/a	28.04	3.67	2.93	0.001
**Zbtb18**	1E-182	-421	0	0.14	0.08	*	66.11	110.90	-0.75	0.008
**Smad3**	1E-169	-390	0	0.54	0.44	*	30.35	15.03	1.01	0.001
**Olig2**	1E-167	-386	0	0.45	0.36	*	24.26	24.21	0.00	0.996
**Pax6**	1E-158	-366	0	0.06	0.02	*	226.36	95.15	1.25	0.001
**Rfx1**	1E-150	-346	0	0.07	0.03	n/a	10.26	11.96	-0.22	0.635
**Pbx3**	1E-126	-290	0	0.08	0.05	n/a	17.60	3.90	2.17	0.001
**Atf1**	1E-115	-266	0	0.17	0.12	n/a	21.63	19.86	0.12	0.840
**Ascl1**	1E-110	-255	0	0.34	0.27	n/a	113.27	220.62	-0.96	0.001
**Rfx5**	1E-104	-240	0	0.09	0.06	n/a	2.81	3.96	-0.49	0.311
**Ehf**	1E-100	-232	0	0.26	0.20	n/a	0.18	0.78	-2.11	0.001
**Mitf**	1E-95	-219	0	0.20	0.15	n/a	0.46	0.31	0.60	0.594
**Myb**	1E-93	-215	0	0.36	0.30	n/a	96.55	70.08	0.46	0.510
**Pknox1**	1E-93	-216	0	0.08	0.04	n/a	14.81	19.31	-0.38	0.303
**Smad2**	1E-90	-209	0	0.31	0.25	n/a	27.38	26.48	0.05	0.938
**Smad4**	1E-86	-199	0	0.31	0.25	n/a	55.15	64.52	-0.23	0.580
**Tcf12**	1E-85	-198	0	0.23	0.18	*	27.45	42.97	-0.65	0.244
**Etv1**	1E-85	-197	0	0.26	0.20	n/a	1.78	5.42	-1.61	0.004
**Tcf3**	1E-81	-189	0	0.08	0.05	*	18.16	11.66	0.64	0.726
**Otx2**	1E-80	-186	0	0.23	0.17	n/a	12.26	51.41	-2.07	0.056
**Zfx**	1E-79	-182	0	0.20	0.15	n/a	3.92	6.00	-0.61	0.440
**Prdm1**	1E-77	-179	0	0.13	0.09	n/a	17.29	49.52	-1.52	0.001
**Tcf4**	1E-74	-171	0	0.14	0.10	n/a	19.15	67.98	-1.83	0.001
**THRa**	1E-72	-167	0	0.09	0.06	n/a	46.29	52.34	-0.18	0.698
**Pbx1**	1E-67	-155	0	0.03	0.02	n/a	5.27	6.34	-0.27	0.839
**Ets1**	1E-66	-152	0	0.20	0.16	n/a	0.91	3.54	-1.95	0.001
**Foxo1**	1E-65	-151	0	0.40	0.34	n/a	4.34	6.67	-0.62	0.069
**Neurod1**	1E-58	-134	0	0.23	0.18	n/a	60.64	218.59	-1.85	0.001

**Tbx20**	1E-58	-135	0	0.06	0.04	n/a	0.62	0.01	5.64	0.027
**Mef2b**	1E-53	-124	0	0.19	0.15	n/a	0.49	0.00	7.18	0.524
**Max**	1E-50	-117	0	0.12	0.09	n/a	21.72	23.55	-0.12	0.867
**Gabpa**	1E-47	-110	0	0.17	0.13	n/a	28.97	34.13	-0.24	0.649
**Clock**	1E-43	-100	0	0.10	0.08	n/a	4.14	13.35	-1.69	0.001
**Atf2**	1E-41	-96	0	0.09	0.06	n/a	10.11	8.92	0.18	0.884
**Atf4**	1E-39	-90	0	0.07	0.05	n/a	218.36	191.76	0.19	0.790
**Foxm1**	1E-39	-91	0	0.24	0.20	*	82.94	88.42	-0.09	0.877
**Maz**	1E-34	-78	0	0.18	0.15	*	300.90	268.31	0.17	0.753
**Foxa1**	1E-34	-79	0	0.27	0.24	n/a	0.03	0.33	-3.26	0.041
**Elk1**	1E-28	-66	0	0.08	0.06	n/a	14.70	15.06	-0.03	0.958
**Elk4**	1E-28	-64	0	0.08	0.06	n/a	6.43	12.28	-0.93	0.001
**Mef2a**	1E-26	-61	0	0.09	0.07	n/a	10.18	9.46	0.11	0.880
**Lhx2**	1E-22	-51	0	0.43	0.40	*	3.76	3.10	0.28	0.909
**Stat3**	1E-22	-51	0	0.10	0.08	n/a	5.85	8.96	-0.62	0.189
**Usf2**	1E-21	-49	0	0.06	0.05	n/a	28.81	34.02	-0.24	0.565
**Usf1**	1E-19	-45	0	0.08	0.06	n/a	36.05	31.68	0.19	0.703
**E2f6**	1E-18	-42	0	0.05	0.04	n/a	32.93	23.41	0.49	0.228
**Nrf1**	1E-17	-40	0	0.01	0.00	n/a	15.87	16.92	-0.09	0.916
**Mef2d**	1E-16	-39	0	0.05	0.04	n/a	14.62	23.25	-0.67	0.025
**Elf1**	1E-16	-39	0	0.07	0.06	n/a	2.64	6.64	-1.33	0.121
**Klf10**	1E-15	-35	0	0.07	0.06	n/a	10.48	18.94	-0.85	0.004
**Sox3**	1E-15	-36	0	0.45	0.43	n/a	15.45	20.11	-0.38	0.345
**E2f4**	1.E-09	-22	0	0.03	0.03	n/a	57.13	45.66	0.32	0.381
**Sp1**	1.E-08	-19	0	0.02	0.01	n/a	29.88	39.14	-0.39	0.224
**JunD**	1.E-08	-19	0	0.02	0.02	n/a	174.51	141.08	0.31	0.434
**E2f1**	1.E-08	-21	0	0.01	0.01	n/a	29.69	59.28	-1.00	0.010
**Klf9**	1.E-08	-20	0	0.05	0.04	n/a	4.21	14.81	-1.82	0.001
**Egr1**	1.E-07	-18	0	0.08	0.07	n/a	288.67	587.92	-1.03	0.005
**MafK**	1.E-07	-17	0	0.05	0.04	*	12.53	7.45	0.75	0.031
**Zbtb33**	1.E-06	-16	0	0.00	0.00	n/a	6.16	9.94	-0.69	0.277
**Klf4**	1.E-05	-12	0	0.04	0.04	n/a	11.12	7.30	0.61	0.098
**Gfy**	1.E-02	-6	0.003	0.01	0.01	*	0.98	0.10	3.25	0.004
**Yy1**	1.E-02	-5	0.006	0.01	0.01	n/a	67.79	58.95	0.20	0.644
**Zbtb12**	1.E-02	-6	0.003	0.06	0.05	n/a	73.24	38.54	0.93	0.001
**E2f7**	1.E-02	-6	0.004	0.01	0.01	n/a	26.28	22.05	0.25	0.532
**Zscan22**	1	-2	0.119	0.01	0.01	n/a	2.98	3.43	-0.21	0.850
**Sox2**	1	-1	0.340	0.27	0.27	n/a	168.62	123.39	0.45	0.196
**Sox4**	1	-1	0.649	0.24	0.24	*	115.68	183.95	-0.67	0.014
**Foxp1**	1	0	1	0.10	0.11	*	17.01	1.81	3.23	0.001
**GLI3**	1	0	1	0.02	0.03	n/a	19.86	42.70	-1.10	0.001

**Sox9**	1	0	0.911	0.23	0.23	*	50.72	83.48	-0.72	0.006
**NF1**	1	0	1.000	0.05	0.13	n/a	9.34	14.75	-0.66	0.024
**Bach1**	1	0	0.796	0.01	0.01	n/a	16.53	13.20	0.32	0.372
**Bach2**	1	0	1	0.04	0.04	n/a	5.01	6.16	-0.30	0.474
**Ctcf**	1	0	1	0.03	0.08	n/a	91.43	101.59	-0.15	0.763

Supplementary Table 2. Transcription factors with known motifs that are enriched in open chromatin regions specific to E14 RPCs

Transcription factors with known motifs that are expressed in early-stage RPCs and overrepresented in open chromatin regions specific to E14 RPCs, are shown. Non-redundant IDs are listed, with the highest RNA-Seq reads counts (FPKM) reported for each transcription factor. Asterisk indicates evidence of expression in the retina (Supplementary Data 1).

Known motifs in P2 RPCs specific open chromatin regions										
	**p-val**	**ln p-val**	**q-val (Benj.)**	**% of Target Seq**	**% of Bckg Seq**	**Expression by ISH, FACS-microrray**	**E14 Chx10-GFP+ve (FPKM)**	**P2 Chx10-GFP+ve (FPKM)**	**log_2FC (FPKM) E14 RPCs vs P2 RPCs**	**q-val E14 vs P2 RPCs**
**Nf1**	1.E-900	-2073	0	0.16	0.06	*	9.34	14.75	-0.66	0.024
**Ctcf**	1.E-743	-1713	0	0.10	0.03	*	91.43	101.59	-0.15	0.763
**Gli3**	1.E-35	-81	0	0.03	0.02	*	19.86	42.70	-1.10	0.001
**Bach2**	1.E-04	-10	0.001	0.04	0.04	n/a	5.01	6.16	-0.30	0.474
**Foxp1**	1.E-02	-6	0.059	0.09	0.09	*	17.01	1.81	3.23	0.001

Supplementary Table 3. Transcription factors with known motifs that are enriched in open chromatin regions specific to P2 RPCs

Known motifs for transcription factors expressed in late RPCs (RNA-Seq) from *Chx10-Cre:eGFP* mice and overrepresented in open chromatin regions exclusive to P2 RPCs (ATAC-Seq) are reported. Asterisks indicate evidence of expression in the retina (Supplementary Data 1). Expression values (log_2FC E14/P2 RPCs; q-val < 0.05) for the nuclear factor 1 (NF-1) family proteins encoded by four different genes (*Nfia, Nfib, Nfic* and *Nfix*) are as follows: -1.92, -3.47, -1.78, -5.59. Non redundant IDs are listed, with the highest RNA-Seq reads counts (FPKM) for any given transcription factor.

	E14 ChIP-Seq	P2 ChIP-Seq	shared in E14 and P2 ChIP-Seq	E14 stage-specific	P2 stage-specific	Total DEG Gene IDs identified by RNA-Seq
**total Peaks**	6270	5970	2135	4135	3854	
**Putatively associated to E14/P2 (GFP+) DEG**	1067 (518 Gene IDs)	1302 (657 Gene IDs)	428 (293 Gene IDs)	652 (380 Gene IDs)	630 (395 Gene IDs)	2679
**Putatively associated to E14(GFP+/-) DEG**	1666 (790 Gene IDs)	1947 (1008 Unique Gene IDs)	336 (215 Gene IDs)	1076 (613 Gene IDs)	930 (619 Gene IDs)	5216
**Putatively associated to P2(GFP+/-) DEG**	1046 (509 Gene IDs)	1243 (667 Unique Gene IDs)	47 (34 Gene IDs)	688 (387 Gene IDs)	635 (417 Gene IDs)	3034
**Putatively associated to early and late RPCs DEG**	2207 (35.2%)	2599 (43.53%)	811(37.98%)	820 (19.83%)	852 (22.11%)	
**Gene IDs associated to TFs with Pioneer potential PIQ > 1**	153 Pks (57 Unique TFs IDs, [26 E14/P2 (GFP+) DEG]	112 Pks (59 Unique TFs IDs, [24 E14/P2 (GFP+) DEG])	30 Pks (19 Unique TFs IDs; [7 E14/P2 (GFP+) DEG])	88 Pks (42 Unique TFs IDs; [17 E14/P2 (GFP+) DEG]	64 Pks (46 Unique TFs IDs, [18 E14/P2 (GFP+) DEG]	

Supplementary Table 4. Assignment of early and late RPCs gene sets to the LHX2 ChIP-Seq repertoire

Differentially expressed genes (DEG) were identified by pairwise comparison of flow sorted E14 and P2 RNA-Seq profiles from *Chx10-Cre:eGFP* mice, and putatively assigned to the closest LHX2 ChIP-Seq peak.

	Total RNA-Seq DEG - Unique Gene IDs	E14 Lhx2 dependent	P2 Lhx2 dependent	Lhx2 dependent unique Gene IDs
**associated to E14/P2 (GFP+) DEG**	2679 (2761 Transcripts)	1129 (E14 GFP+: 46.39% down in E14 cKO); (P2 GFP+: 66.13% up in E14 Lhx2 cKO)	147 (E14 GFP+: 68.57% down in P2 Lhx2 cKO); (P2 GFP+: 27.09% down in P2 Lhx2 cKO)	1270 (47.41%)
**associated to E14 (GFP+/-) DEG**	5216 (5640 Transcripts)	1975 (E14 GFP+: 46.87% down in E14 Lhx2 cKO); (E14 GFP-: 81.44% up in E14 Lhx2 cKO)	334 (E14 GFP+: 53.59% down in P2 Lhx2 cKO); (E14 GFP-: 54.70% up in P2 Lhx2 cKO)	1985 (38.06 %)
**associated to P2 (GFP+/-) DEG**	3034 (3141 Transcripts)	1317 (P2 GFP+:35.89% up in E14 Lhx2 cKO) (P2 GFP-: 84.12% up in E14 Lhx2 cKO)	289 (P2 GFP+: 35.51% down in P2 Lhx2 cKO) (P2 GFP-: 55.15% up in P2 Lhx2 cKO)	1324 (43.64%)
**Total Unique DEG IDs**	7038			2654 (37.71%)

Supplementary Table 5. Dependence of early and late RPCs transcriptome on *Lhx2* expression

Differentially expressed genes (DEG) were identified from pairwise comparison of flow sorted E14 and P2 RNA-Seq profiles from *Chx10-CreGFP* mice. *Lhx2*-dependent genes exhibit variation in least one developmental time point by pairwise comparison of RNA-Seq from age-matched control and *Lhx2* cKO conditions (q-val < 0.05 unless indicated otherwise).

Co-occurrence	E14 Lhx2 ChIP-Seq	P2 Lhx2 ChIP-Seq	ATAC-Seq coverage at Lhx2 targets	Age-matched ATAC-Seq control	Age-matched ATAC-Seq Lhx2 cKO	Variation in local coverage
E14 ATAC-Seq Chx10 GFP+	4.25% (0.24% centered); ln p-val Obs./Exp = -9606	ND	E14 ATAC-Seq Chx10 GFP+	$R^2 = 0.76$	$R^2 = 0.37$	*p-val* < 0.0001
P2 ATAC-Seq Chx10 GFP+	ND	3.48% (0.14% centered); ln p-val Obs./Exp. = -7609	P2 ATAC-Seq Chx10 GFP+	R^2=0.88	R^2=0.87	*p-val* < 0.0001
P2 ATAC-Seq Chx10 GFP-	ND	2% (0.09% centered); ln p-val Obs./Exp. = -4107	P2 ATAC-Seq Chx10 GFP-	R^2=0.897	$R^{2=}$0.893	p-val < 0.0001

Genes identified in modules of coordinately accessible chromatin	E14 Lhx2 ChIP-Seq	P2 Lhx2 ChIP-Seq
E14 ATAC-Seq Chx10 GFP+	Fem1b,Ndrg1,Prox1,Usp44,Aurka,Cdk5rap2,Gsg2,Anapc15-ps,Mif,Snai2,Zfp385a,Snai1,Usp47,Arid1a,Chd3,Smarca2,Smarca5,Baz1a,Cecr2,Csnk2a1,Mbd3,Actl6a,Hdac1,Chaf1a,Chd4,Ep300,Hes1,Hes5,Jag1,Kat2b,Maml1,Ncor2,Notch1,Lfng,Notch2,Maml2A,hctf1A,lms1,Aurkb,Brca1,Bub1b,Casc5,Ccnd1,Cdc6,Cdk1,Ckap5,Dync1h1,Ercc6l,Hist1h2bg,Hist1h2bj,Hist1h2bk,Hist1h2bm,Hist1h4i,Kif18a,Lmna,Ndc80,Ninl,Pcnt,Pola1,Pole,Pttg1,Ranbp2,Rfc5,Ruvbl2,Syne2,Taok1,Tubb4a,Ube2d1,Hist1h4h,Mis18bp1,Ppp2r5e,Prim1,Rpa2,Chek1,Hist1h2ae,Mcm3,Stag1,Rangap1,Dna2,Fbxo5,Sgol1,Psme4,Ruvbl1,Rfc2,Hist1h2bb,Pold3,Cenpi,Hist1h2ab,Hist4h4,Psmb3,Gmnn,Sgol2,Smc3,Kif23,Gins4,Ccnb2,Pole2,Mad1l1,Wrap53,Mis18a,Psmd14,Nup107,Cep76,Mybl2,Cep135,Dyrk1a,Psmb2,Rfc3,Bub1,Cenpc1,Cdc25c,Dsn1,Psmd11,Tgfb3,Tipin,Pds5a,Wapal,Fbxw7,Gtpbp4,Mapk15,	
P2 ATAC-Seq Chx10 GFP+		Cdk6,Gas1,Pla2g16,Rhob,Cdkn1c,Phf20,Taf6,Kansl1,Max,Ei24,Chd3,Mbd3,Hdac1,Chd4,Gatad2a,Rbbp7,Foxn4,Hmga2,Prdm5,Sox2,Zfp703,Lef1,Gmnn,Tob2,Pbx1,Kat6b,Notch2,Asxl1,Neurod1,Hdac5,H2afx,Hist1h2bg,Hist1h2bj,Hist1h2bk,Hist1h2br,Hist1h3a,Hist1h3c,Hist1h3e,Hist1h3g,Hist1h4i,Hist1h4k,Hist2h2ac,Hist2h2bb,Hist2h3c2,Hist3h2a,Hist3h2ba,Hist1h3i,Hist1h4h,Hist1h2ae,Hist1h2bh,Hist1h2bb,Hist1h2ak,Hist1h2ab,Hist4h4,H2afz,Sos1,Hist1h2ah,Hist1h4c,Hist1h3d,Hist2h4,Prdm8,Setd1a,Phf1,Taf1,Ruvbl1,Cbx5,Actb,Nsd1,Kat8,Stk38,

Supplementary Table 6. Fraction of open chromatin regions overlapping with LHX2 age-matched peaks, related variations in local coverage and gene expression in age-matched *Lhx2* cKO conditions

(6.1). High confidence peaks with the highest differential in accessibility between nucleosomes units and flanking nucleosomes free, transposable regions, and a minimum distance of 300bp

were identified from two ATAC-Seq replicates. ATAC-Seq coverage estimated upon library size normalization. Two tailed t-test comparisons as indicated. High confidence ChIP-Seq peaks were called from a minimum of two normalized replicates. Co-occurrence of ATAC-Seq regions and age-matched LHX2 ChIP-Seq targets was determined by hypergeometric test in E14 *Chx10-Cre* whole retina (control), *Chx10-Cre; Lhx2* $^{lox/lox}$ whole retina (E14 *Lhx2* cKO) and also in P0 electroporated, P2 harvested pCAG-*Cre: GFP* (P2 control) and pCAG-*Cre:GFP; Lhx2* $^{lox/lox}$ (P2 *Lhx2* cKO) conditions. Centrality allowed within 20bp distance between summits. **(6.2).** Genes found in modules of coordinately accessible chromatin and dependent on LHX2 (RNA-Seq from E14, P2 *Lhx2* cKO) are also reported. Functional gene ontologies from which the genes were extracted include chromatin modifying factors, genes involved in cell cycle regulation, DNA replication, G2/M checkpoints and retinal phenotypes.

	E14 ATAC-Seq GFP+	E14 ATAC-Seq control	E14 ATAC-Seq cKO	P2 ATAC-Seq GFP+	P2 ATAC-Seq GFP-	P2 ATAC-Seq control	P2 ATAC-Seq cKO
**E14 Lhx2 ChIP-Seq**	76.67%	73.72%	34.47%	ND	ND	ND	ND
**P2 Lhx2 ChIP-Seq**	ND	ND	ND	61.72%	48.70%	61.24%	53.00%
**ln p-val Obs/Exp.**	-9605	-9051	-4050	-7609	-4108	-8745	-7876

Supplementary Table 7. Chromatin accessibility at the LHX2 target sites

High confidence peaks with the highest differential in accessibility between nucleosomes units and flanking nucleosomes free, transposable regions, and a minimum distance of 300bp were identified from two ATAC-Seq replicates. High confidence peaks were called from a minimum of two ChIP-Seq normalized replicates. Co-occurrence of ChIP-Seq peaks and age-matched open chromatin regions was determined by hypergeometric test in E14 *Chx10-Cre* whole retina (control), *Chx10-Cre; Lhx2* $^{lox/lox}$ whole retina (E14 *Lhx2* cKO) and also in P0 electroporated, P2 harvested pCAG-*Cre: GFP* (P2 control) and pCAG-*Cre:GFP; Lhx2* $^{lox/lox}$ (P2 *Lhx2* cKO) conditions.

	E14 ATAC-Seq GFP+	E14 ATAC-Seq control	E14 ATAC-Seq Lhx2 cKO	P2 ATAC-Seq GFP+	P2 ATAC-Seq control	P2 ATAC-Seq Lhx2 cKO
**# Open chromatin regions**	113004	103736	54029	106014	81373	63330
**Percentage loss in Lhx2 cKO**	52.19%	47.92%	ND	40.26%	22.17%	ND

Supplementary Table 8. Global variations in chromatin accessibility following *Lhx2* loss-of-function

High confidence peaks with the highest differential in accessibility between nucleosomes units and flanking nucleosomes free, transposable regions, and a minimum distance of 300bp were identified from two ATAC-Seq replicates. Percentage of loss in accessibility is reported for *Chx10-Cre; Lhx2* $^{lox/lox}$ whole retina (E14 *Lhx2* cKO) compared to E14 control condition (*Chx10-Cre* whole retina or GFP+ sorted fraction) and from P0 electroporated, P2 harvested pCAG-*Cre:GFP; Lhx2* $^{lox/lox}$ (P2 *Lhx2* cKO) versus pCAG-*Cre:GFP* (P2 control) or *Chx10-Cre GFP+* flow sorted fraction.

	E14 ChIP-Seq Peaks	P2 ChIP-Seq	shared in E14 and P2 ChIP-Seq Peaks	E14 stage-specific Peaks	P2 stage-specific
**Lhx2 dependent**	1540 (736 Unique Gene IDs)	398 (185 Unique Gene IDs, p val < 0.05; 78 Unique Gene IDs, q val < 0.05)	532 (354 Unique Gene IDs)	1095 Pks (642 unique gene IDs)	211 Pks (130 Unique Gene IDs, p val < 0.05)
**Lhx2 dependent, E14/P2 (GFP+) DEG associated**	770 (275 Unique Gene IDs)	178 (84 Unique Gene IDs, p val < 0.05)	132 Unique Gene IDs (37.29%)	157 Unique gene IDs (24.45%)	45 Unique Gene IDs (p-val < 0.05)
**Lhx2 dependent, all retinal E14; P2 DEG associated**	770 (50%)	311 (P2 cKO p-val < 0.05; 12 P2 cKO q-val < 0.05) (78.14%)	238 Unique Gene IDs (67.23%)	357 Unique Gene IDs (55.61%)	75 unique Gene IDs (p-val < 0.05)
**Lhx2 dependent TFs IDs with Pioneer potential PIQ > 1**	31 Unique TFs [16 E14/P2 (GFP+) DEG]	2 Unique TFs [4 TFs P2 cKO p-val < 0.050; none E14/P2 (GFP+) DEG]	30 Pks;18 Unique TFs [17 in E14 cKO; 1 in P2 cKO; 7 E14/P2 (GFP+) DEG]	88 Pks; 23 Unique TFs [12 E14/P2 (GFP+) DEG]	64 Pks; 1 Unique TFs (2 TFs P2 cKO p-val < 0.05); [1 E14/P2 (GFP+) DEG]
**Likely functional Peaks**	1540 (24.56%)	2685 (44.97%)			

Supplementary Table 9. Functional assignment of LHX2 cis-regulatory elements

Lhx2-dependent peaks were defined as cis-regulatory regions whose closest gene exhibits variations in gene expression by RNA-Seq in the age-matched *Lhx2* cKO (q-val < 0.05 unless indicated otherwise). Differentially expressed genes (DEG) were identified from pairwise comparison of flow sorted retinal E14 and P2 RNA-Seq profiles from *Chx10-CreGFP* mice (q-val < 0.05). The number of assigned transcription factors with Pioneering Protein Interaction quantitation (PIQ) > 1 and variations in gene expression by RNA-Seq in age-matched *Lhx2* cKO is reported. Functional Peaks were defined as LHX2 bound cis-regulatory regions that exhibit variations in local coverage by ATAC-Seq in age-matched *Lhx2* cKO and for which the closest, putatively related gene satisfies at least one of the following conditions: variation in gene expression in age-matched *Lhx2* cKO and/or variation in gene expression across development (E14/P2 GFP+).

# of footprints	E14 Chx10-GFP+	P2 Chx10-GFP+	E14 whole retina	E14 Chx10-GFP; Lhx2 fl/fl	P2 pCAG-GFP	P2 pCAG-GFP;Lhx2 fl/fl	E14 Percentage loss	P2 Percentage loss
**Lhx2**	3223	4180	2438	98	2907	1772	95.98	39.04
**NF-I**	5836	16601	4991	779	11656	10866	84.39	6.78
**Sox2**	7906	8950	6353	443	5987	5008	93.03	16.35
**Klf4**	25121	43784	25707	3186	21824	21067	87.61	3.47
**Ascl1**	681	1342	606	41	424	416	93.23	1.89
**Hes5**	803	1493	751	74	594	603	90.15	-1.52

Supplementary Table 10. Footprint counts for transcription factors expressed in early and late-stage RPCs are reduced following loss of LHX2 function

Total footprint counts are listed in open chromatin regions detected in E14 and P2 RPCs, age-matched controls and *Lhx2* cKO samples.

	E14 Chx10-Cre vs Chx10-Cre;Lhx2 fl/fl	P2 elec. pCAG-CreGFP vs elec. pCAG-CreGFP;Lhx2 fl/fl
**Lhx2**	5.10E-208	1.09E-59
**NFI**	1.00E-208	4.06E-85
**Sox2**	1.00E-208	1.00E-208
**KLF**	1.00E-208	4.09E-122
**Ascl1**	7.72E-04	4.39E-150
**Hes5**	1.90E-27	1.34E-121
**Smad3**	1.00E-208	1.10E-03
**Max**	1.00E-208	7.11E-44
**Zbtb33**	6.86E-193	1.00E-208
**Zfx**	1.00E-208	1.00E-208
**Gabpa**	1.00E-208	6.38E-90
**Neurod1**	1.00E-208	1.00E-208
**Pknox1**	2.08E-23	1.00E-208
**Tgif1**	1.00E-208	1.00E-208
**Etv1**	1.00E-208	1.21E-05
**Etv5**	1.00E-208	3.45E-06

Supplementary Table 11. Statistical test for variation in transcription factors footprints observed in *Lhx2* cKO samples

T-test is shown for mean scores within 200 bp from footprint center. Reported are putative LHX2 co-factors for which a co-occurrent ChIP-Seq motif could be found in at least one developmental time point and for which a high pioneering protein interaction quantitation (PIQ) could be predicted (Supplementary Table 12). LHX2 footprints were also identified in proximity to its own *Lhx2* promoter (mm9, chr2: 38,203,496-38,203,526; chr2: 38,203,568-38,203,598; chr2: 38,203,570-38,203,600; chr2: 38,213,539-38,213,569).

Predicted Pioneer	E14 Chx10-GFP+ (FPKM)	P2 Chx10-GFP+ (FPKM)	log2 FC (FPKM) Chx10-GFP+ E14/P2	q-value Chx10-GFP+ E14/P2	Log Odd (PIQ score)	log2 FC (FPKM) E14 Lhx2 cKO/Ctrl	E14 Lhx2 cKO/Ctrl (FPKM) q value	log_2 FC ATAC-Seq (Fraction Reads/10 Mio) E14 Lhx2 cKO/Ctrl	q-val co-occurring motifs by E14 Lhx2 ChIP-Seq	log_2 FC (FPKM) P2 Lhx2 cKO/Ctrl	P2 Lhx2 cKO/Ctrl (FPKM) p-value	log_2 FC ATAC-Seq (Fraction Reads/10 Mio) P2 Lhx2 cKO/WT	q-val co-occurring motifs by P2 Lhx2 ChIP-Seq
Arnt	17.38	19.42	-0.16	8.10E-01	28.03	n/a	n/a	-1.97	n/a	n/a	n/a	n/a	n/a
Ascl1	113.27	220.62	-0.96	8.41E-04	18.79	n/a	n/a	-2.29	0.00E+00	n/a	n/a	n/a	0.00E+00
**Atf1**	21.63	19.86	0.12	8.40E-01	19.16	0.67	4.19E-02	-2.74	0.00E+00	n/a	n/a	n/a	0.00E+00
Atoh7	331.83	10.40	5.00	8.41E-04	9.38	-1.34	6.10E-04	-2.29	n/a	n/a	n/a	n/a	n/a
Atoh8	21.89	8.13	1.43	8.41E-04	-5.34	-0.99	7.99E-03	-2.58	n/a	-0.95	2.38E-02	n/a	n/a
**Bbx**	10.03	15.00	-0.58	3.83E-02	-4.56	-0.90	6.10E-04	-1.07	n/a	n/a	n/a	n/a	n/a
Brca1	22.65	20.52	0.14	7.85E-01	-0.47	-1.59	6.10E-04	n/a	n/a	n/a	n/a	n/a	n/a
Btd	8.64	7.47	0.21	7.62E-01	59.13	n/a	n/a	n/a	n/a	n/a	n/a	n/a	n/a
**Creb1**	18.71	24.58	-0.39	2.87E-01	30.32	-0.63	4.65E-02	n/a	n/a	n/a	n/a	-1.44	n/a
**Crx**	28.60	158.24	-2.47	8.41E-04	5.31	-1.60	6.10E-04	-2.60	0.00E+00	0.55	4.75E-03	n/a	0.00E+00
**Ctcf**	91.43	101.59	-0.15	7.63E-01	31.42	n/a	n/a	n/a	n/a	n/a	n/a	n/a	0.00E+00
**Cux1**	27.35	26.49	0.05	9.77E-01	-10.32	n/a	n/a	-1.87	n/a	-1.23	1.88E-02	n/a	n/a
Deaf1	17.61	14.64	0.27	7.01E-01	18.47	0.82	3.87E-02	-0.48	n/a	n/a	n/a	n/a	n/a
**Dlx2, Dlx2**	26.21	1.97	3.73	8.41E-04	2.86	n/a	n/a	-2.07	n/a	1.10	3.54E-02	0.65	n/a
**E2f1**	29.69	59.28	-1.00	9.59E-03	35.28	n/a	n/a	-1.85	n/a	n/a	n/a	0.25	n/a
E2f2	13.59	15.29	-0.17	7.39E-01	63.23	n/a	n/a	-2.02	n/a	n/a	n/a	n/a	n/a
**E2f3**	17.10	9.06	0.92	4.63E-03	60.63	n/a	n/a	-2.89	n/a	n/a	n/a	n/a	n/a
**Egr1**	288.67	587.92	-1.03	4.63E-03	38.34	3.96	6.10E-04	-3.08	0.00E+00	n/a	n/a	n/a	0.00E+00
**Elk1**	14.70	15.06	-0.03	9.58E-01	35.88	n/a	n/a	-2.78	0.00E+00	n/a	n/a	n/a	0.00E+00
Elk4	6.43	12.28	-0.93	8.41E-04	37.62	n/a	n/a	-2.29	0.00E+00	n/a	n/a	n/a	0.00E+00
**Esrra**	14.87	11.31	0.40	4.81E-01	4.79	n/a	n/a	n/a	n/a	n/a	n/a	n/a	n/a
Ets2	11.26	11.95	-0.09	9.00E-01	30.57	1.20	6.10E-04	-1.12	n/a	n/a	n/a	n/a	n/a
**Etv5**	47.77	71.29	-0.58	3.52E-02	30.57	-1.52	6.10E-04	-2.31	n/a	n/a	n/a	n/a	n/a
Fezf2	43.91	6.49	2.76	8.41E-04	-8.10	n/a	n/a	-2.13	n/a	n/a	n/a	n/a	n/a
Fhl1	11.41	8.73	0.39	8.22E-01	20.22	n/a	n/a	n/a	n/a	-0.68	2.28E-02	n/a	n/a
**Fos**	242.76	568.56	-1.23	8.41E-04	1.86	2.04	7.68E-03	-1.74	n/a	n/a	n/a	n/a	n/a
**Fosb**	81.77	95.71	-0.23	6.51E-01	17.53	n/a	n/a	-2.88	n/a	-0.71	1.55E-03	n/a	n/a
Foxd1	17.69	7.80	1.18	8.41E-04	-3.41	n/a	n/a	-2.09	n/a	n/a	n/a	n/a	n/a
Foxj3	13.72	12.47	0.14	8.64E-01	-15.95	n/a	n/a	-3.03	n/a	n/a	n/a	n/a	n/a
Foxk1	11.74	11.24	0.06	9.23E-01	-17.18	1.07	6.10E-04	-3.08	n/a	n/a	n/a	n/a	n/a
Foxk2	34.19	31.28	0.13	8.09E-01	-2.54	n/a	n/a	n/a	n/a	n/a	n/a	n/a	n/a
Foxn2	10.82	17.45	-0.69	1.63E-02	-15.82	n/a	n/a	-2.15	n/a	n/a	n/a	n/a	n/a
Foxo3	21.00	33.34	-0.67	1.79E-02	-2.39	-0.63	4.72E-02	-2.75	n/a	n/a	n/a	n/a	n/a
Foxp4	23.56	8.03	1.55	8.41E-04	-2.14	1.42	6.10E-04	-3.15	n/a	n/a	n/a	n/a	n/a
**Gabpa**	28.97	34.13	-0.24	6.49E-01	35.48	n/a	n/a	-4.31	0.00E+00	n/a	n/a	n/a	0.00E+00
**Glis2**	35.67	32.69	0.13	8.18E-01	5.53	0.60	4.74E-02	n/a	n/a	n/a	n/a	n/a	n/a
Gmeb1	13.14	12.99	0.02	9.81E-01	20.30	n/a	n/a	n/a	n/a	n/a	n/a	n/a	n/a

Hap1	17.06	18.78	-0.14	8.47E-01	20.19	n/a	n/a	n/a	n/a	n/a	n/a	n/a	n/a
**Hbp1**	35.45	53.46	-0.59	7.30E-02	-0.86	n/a	n/a	n/a	n/a	n/a	n/a	n/a	n/a
Hes5	342.85	354.94	-0.05	9.37E-01	31.83	-1.62	6.10E-04	-2.57	n/a	n/a	n/a	n/a	n/a
Hey1	43.57	31.27	0.48	1.12E-01	25.18	-0.97	1.58E-03	-2.92	n/a	n/a	n/a	n/a	n/a
Heyl	16.47	45.79	-1.48	8.41E-04	37.40	1.44	6.10E-04	n/a	n/a	n/a	n/a	n/a	n/a
Hltf	6.62	9.09	-0.46	3.83E-01	-12.00	n/a	n/a	-1.34	n/a	n/a	n/a	n/a	n/a
**Hmbox1**	14.03	19.23	-0.46	1.86E-01	-10.34	n/a	n/a	-1.45	n/a	n/a	n/a	n/a	n/a
**Hmx1;**<br>**Hmx1**	79.23	37.03	1.10	8.41E-04	3.32	-2.07	6.10E-04	-1.36	n/a	n/a	n/a	n/a	n/a
Hsf1	30.95	23.95	0.37	3.19E-01	-3.30	n/a	n/a	-2.07	n/a	n/a	n/a	n/a	n/a
Id1	248.57	113.87	1.13	8.41E-04	-7.29	n/a	n/a	-2.63	n/a	n/a	n/a	n/a	n/a
Insm1	89.62	58.13	0.62	2.11E-02	15.78	0.67	3.12E-02	n/a	n/a	n/a	n/a	-1.37	n/a
Irf1	9.60	7.45	0.37	4.90E-01	-2.40	n/a	n/a	-2.99	0.00E+00	1.07	7.50E-04	n/a	n/a
Irf3	14.87	16.94	-0.19	7.86E-01	6.69	n/a	n/a	n/a	n/a	n/a	n/a	n/a	n/a
**Klf11**	9.84	8.36	0.23	6.38E-01	52.78	n/a	n/a	-1.83	n/a	n/a	n/a	n/a	n/a
Klf13	19.08	52.01	-1.45	8.41E-04	21.52	n/a	n/a	-2.68	n/a	n/a	n/a	n/a	n/a
Klf4	11.12	7.30	0.61	9.80E-02	42.31	2.27	6.10E-04	-2.21	n/a	n/a	n/a	1.14	n/a
**Klf7**	12.86	5.80	1.15	5.69E-03	52.37	n/a	n/a	-1.97	n/a	n/a	n/a	n/a	n/a
**Lhx2;**<br>**Lhx2**	152.71	183.84	-0.27	5.09E-01	4.62	3.20	3.18E-02	-2.56	0.00E+00	n/a	n/a	n/a	0.00E+00
**Mafk**	12.53	7.45	0.75	3.11E-02	-9.00	n/a	n/a	-2.37	2.76E-02	n/a	n/a	n/a	n/a
Max	21.72	23.55	-0.12	8.67E-01	20.00	n/a	n/a	n/a	0.00E+00	n/a	n/a	n/a	4.70E-02
**Meis1**	57.05	37.44	0.61	3.86E-02	3.20	-0.58	5.16E-02	-2.52	0.00E+00	n/a	n/a	n/a	0.00E+00
**Meis3**	17.45	10.22	0.77	6.43E-02	4.43	n/a	n/a	-2.88	n/a	n/a	n/a	n/a	n/a
Myb	96.55	70.08	0.46	5.10E-01	7.80	-1.95	6.10E-04	-0.58	0.00E+00	n/a	n/a	n/a	0.00E+00
Mybl1	22.68	69.72	-1.62	8.41E-04	4.56	-1.63	2.44E-03	-2.72	n/a	0.64	3.28E-02	n/a	n/a
Mycn	69.73	41.24	0.76	1.57E-03	28.03	-1.10	6.10E-04	n/a	n/a	n/a	n/a	n/a	n/a
Neurod1	60.64	218.59	-1.85	8.41E-04	15.29	n/a	n/a	-2.24	0.00E+00	0.54	5.35E-03	n/a	0.00E+00
Neurod4	23.06	251.40	-3.45	8.41E-04	16.23	-1.13	2.44E-03	-3.34	n/a	n/a	n/a	n/a	n/a
**Nfia**	2.02	7.66	-1.92	8.41E-04	17.52	3.87	6.10E-04	-2.51	0.00E+00	n/a	n/a	-1.63	0.00E+00
**Nfib**	2.38	26.36	-3.47	8.41E-04	15	2.85	6.10E-04	-3.15	0.00E+00	n/a	n/a	-0.60	0.00E+00
**Nfic**	2.61	9.00	-1.78	8.41E-04	11.42	2.39	6.10E-04	n/a	0.00E+00	n/a	n/a	n/a	0.00E+00
**Nfix**	0.94	45.49	-5.59	8.41E-04	11.76	n/a	n/a	n/a	0.00E+00	n/a	n/a	n/a	0.00E+00
Nfil3	31.13	30.99	0.01	9.91E-01	-12.11	n/a	n/a	-2.66	n/a	n/a	n/a	n/a	n/a
Nfkb1	15.15	13.67	0.15	7.84E-01	18.49	n/a	n/a	-3.15	n/a	n/a	n/a	0.80	n/a
Nr1d2	15.74	33.54	-1.09	8.41E-04	0.77	-0.81	1.16E-02	-1.97	n/a	n/a	n/a	n/a	n/a
**Nr2f1**	102.80	40.41	1.35	8.41E-04	-12.58	0.61	3.63E-02	-2.94	n/a	n/a	n/a	n/a	n/a
**Nr2f2**	11.23	10.31	0.12	8.47E-01	18.26	2.78	6.10E-04	-2.61	n/a	n/a	n/a	n/a	n/a
Nr2f6	41.85	16.26	1.36	8.41E-04	-1.73	n/a	n/a	n/a	n/a	n/a	n/a	n/a	n/a
Nr3c1	12.76	15.51	-0.28	4.73E-01	-7.73	n/a	n/a	-2.39	n/a	n/a	n/a	n/a	n/a
Olig2	24.26	24.21	0.00	9.96E-01	5.03	n/a	n/a	-1.16	0.00E+00	n/a	n/a	n/a	0.00E+00
**Otx2**	78.50	381.74	-2.28	8.41E-04	-3.24	n/a	n/a	n/a	0.00E+00	n/a	n/a	n/a	0.00E+00
**Pax6;**<br>**Pax6**	226.36	95.15	1.25	8.41E-04	0.85	-0.83	4.71E-02	-2.78	0.00E+00	n/a	n/a	0.11	0.00E+00

**Pbx1**	25.59	18.99	0.43	2.64E-01	-9.34	2.44	2.49E-02	-2.77	0.00E+00	-1.01	2.96E-02	-0.70	1.00E-04
Pbx2	68.25	42.95	0.67	5.69E-03	-11.72	n/a	n/a	-2.41	n/a	n/a	n/a	n/a	n/a
Pknox1	14.81	19.31	-0.38	3.03E-01	7.46	n/a	n/a	-1.31	0.00E+00	n/a	n/a	n/a	0.00E+00
Plagl1	275.31	143.34	0.94	2.24E-03	38.05	n/a	n/a	-2.09	n/a	n/a	n/a	n/a	n/a
**Pou2f1; Pou2f1**	5.19	5.83	-0.17	8.06E-01	-9.05	n/a	n/a	-2.99	n/a	n/a	n/a	-0.55	n/a
**Pou3f2**	6.53	18.61	-1.51	8.41E-04	-0.73	n/a	n/a	-2.22	n/a	n/a	n/a	0.40	n/a
Pou6f1	5.06	13.05	-1.37	8.41E-04	9.57	1.82	6.10E-04	-2.52	n/a	n/a	n/a	n/a	n/a
Prdm1	17.29	49.52	-1.52	8.41E-04	-0.48	-1.67	6.10E-04	-2.48	0.00E+00	n/a	n/a	n/a	0.00E+00
Prox1	15.87	43.50	-1.45	8.41E-04	14.24	-1.25	6.10E-04	n/a	n/a	n/a	n/a	-0.04	n/a
**Rara**	7.07	12.16	-0.78	1.88E-01	19.71	n/a	n/a	-2.03	n/a	n/a	n/a	n/a	n/a
**Rax; Rax**	280.78	217.68	0.37	2.89E-01	8.43	-1.93	6.10E-04	n/a	n/a	-0.42	3.39E-02	n/a	n/a
Rela	17.81	19.46	-0.13	8.28E-01	9.16	0.73	2.63E-02	n/a	n/a	n/a	n/a	n/a	n/a
**Rest**	18.16	14.64	0.31	4.07E-01	12.52	-0.68	1.83E-02	n/a	n/a	n/a	n/a	n/a	n/a
Rfx3	7.46	8.53	-0.19	7.55E-01	11.08	n/a	n/a	-2.77	n/a	n/a	n/a	n/a	n/a
Rreb1	5.33	11.24	-1.08	8.41E-04	-3.64	1.53	6.10E-04	-1.86	n/a	n/a	n/a	n/a	n/a
Rxra	14.37	15.18	-0.08	9.14E-01	0.97	n/a	n/a	n/a	n/a	n/a	n/a	n/a	n/a
Six3	119.48	103.23	0.21	6.28E-01	-10.48	2.44	6.10E-04	-1.30	n/a	n/a	n/a	n/a	n/a
Six5	22.47	8.56	1.39	8.41E-04	10.86	n/a	n/a	-2.92	n/a	-1.12	3.50E-03	n/a	n/a
Six6	65.32	41.36	0.66	1.14E-02	-12.54	-2.75	6.10E-04	-2.17	n/a	n/a	n/a	n/a	n/a
**Smad3**	30.35	15.03	1.01	8.41E-04	60.76	n/a	n/a	-1.88	0.00E+00	n/a	n/a	n/a	0.00E+00
**Sox11**	71.94	37.97	0.92	8.41E-04	-7.24	-0.83	9.56E-03	-2.34	n/a	n/a	n/a	n/a	n/a
**Sox12**	35.95	15.44	1.22	8.41E-04	-2.71	1.54	6.10E-04	n/a	n/a	n/a	n/a	n/a	n/a
**Sox13**	14.37	23.21	-0.69	1.93E-02	0.58	0.74	3.35E-02	n/a	n/a	0.71	8.00E-03	n/a	n/a
**Sox2**	168.62	123.39	0.45	1.96E-01	-2.95	-1.41	6.10E-04	-2.62	0.00E+00	n/a	n/a	-0.61	0.00E+00
**Sox4**	115.68	183.95	-0.67	1.44E-02	-5.99	0.85	2.84E-03	-2.20	0.00E+00	n/a	n/a	n/a	0.00E+00
**Sox8**	20.75	159.31	-2.94	8.41E-04	-9.08	n/a	n/a	-2.68	n/a	n/a	n/a	n/a	n/a
**Sox9**	50.72	83.48	-0.72	6.20E-03	4.64	1.36	6.10E-04	n/a	0.00E+00	n/a	n/a	n/a	0.00E+00
**Sp1**	29.88	39.14	-0.39	2.24E-01	22.83	n/a	n/a	-1.97	n/a	n/a	n/a	n/a	n/a
**Sp4**	10.46	11.89	-0.18	6.85E-01	55.22	-0.92	6.10E-04	n/a	n/a	n/a	n/a	n/a	n/a
**Srf**	117.03	67.50	0.79	2.87E-03	-1.74	n/a	n/a	n/a	n/a	n/a	n/a	n/a	n/a
Stat3	5.85	8.96	-0.62	1.90E-01	2.46	n/a	n/a	-3.00	0.00E+00	0.93	1.39E-02	n/a	0.00E+00
**Tbp**	33.39	36.88	-0.14	7.95E-01	4.79	n/a	n/a	-1.50	n/a	n/a	n/a	n/a	n/a
Tcf3	18.16	11.66	0.64	7.26E-01	-8.45	n/a	n/a	-3.01	0.00E+00	n/a	n/a	n/a	0.00E+00
Tcf4	19.15	67.98	-1.83	8.41E-04	-0.27	0.99	7.68E-03	-1.86	0.00E+00	n/a	n/a	n/a	0.00E+00
Tead1	14.82	22.96	-0.63	3.52E-02	7.52	1.50	6.10E-04	n/a	n/a	n/a	n/a	n/a	n/a
Tgif1	30.61	27.95	0.13	8.57E-01	9.06	n/a	n/a	-2.02	0.00E+00	n/a	n/a	n/a	0.00E+00
**Tgif2**	41.41	33.85	0.29	4.70E-01	4.88	n/a	n/a	-0.51	0.00E+00	n/a	n/a	n/a	0.00E+00
Ttk	36.62	39.01	-0.09	8.76E-01	-0.17	-0.95	2.44E-03	n/a	n/a	n/a	n/a	n/a	n/a
Usf1	36.05	31.68	0.19	7.03E-01	20.92	n/a	n/a	-1.47	0.00E+00	n/a	n/a	n/a	n/a
Vax2	43.52	21.23	1.04	1.67E-02	11.02	-1.11	1.07E-02	-3.18	n/a	n/a	n/a	n/a	n/a
**Vsx2**	243.37	209.98	0.21	7.40E-01	5.57	-3.27	6.10E-04	-0.68	n/a	n/a	n/a	n/a	n/a

**Yy1**	67.79	58.95	0.20	6.44E-01	-3.01	n/a	n/a	-2.34	n/a	n/a	n/a	n/a	n/a
Zbtb12	73.24	38.54	0.93	8.41E-04	-4.50	n/a	n/a	n/a	n/a	n/a	n/a	n/a	n/a
Zbtb18	66.11	110.90	-0.75	7.69E-03	-0.18	n/a	n/a	-2.56	0.00E+00	n/a	n/a	-1.35	0.00E+00
Zbtb33	6.16	9.94	-0.69	2.77E-01	14.39	1.42	4.33E-03	n/a	n/a	0.70	2.99E-02	n/a	n/a
Zbtb6	9.69	10.18	-0.07	9.17E-01	6.24	n/a	n/a	-3.64	n/a	n/a	n/a	n/a	n/a
**Zeb1**	17.99	17.45	0.04	9.46E-01	-6.48	n/a	n/a	n/a	n/a	n/a	n/a	n/a	n/a
**Zfp105**	21.39	26.68	-0.32	4.41E-01	-10.73	-0.75	2.25E-02	-2.02	n/a	n/a	n/a	n/a	n/a
**Zfp281**	12.59	13.06	-0.05	9.47E-01	18.74	n/a	n/a	n/a	n/a	n/a	n/a	n/a	n/a
**Zfp410**	32.25	33.52	-0.06	9.36E-01	-8.36	n/a	n/a	-2.11	n/a	n/a	n/a	n/a	n/a
Zfp740	40.31	41.82	-0.05	9.50E-01	18.58	n/a	n/a	-0.69	n/a	n/a	n/a	n/a	n/a
**Zfx**	9.72	11.89	-0.29	5.82E-01	37.81	-0.91	8.63E-03	n/a	0.00E+00	n/a	n/a	n/a	0.00E+00
**Zic1**	52.22	45.85	0.19	6.75E-01	42.79	n/a	n/a	n/a	n/a	n/a	n/a	n/a	n/a
**Zic2**	66.62	43.00	0.63	1.87E-02	41.08	n/a	n/a	n/a	n/a	n/a	n/a	n/a	n/a

Supplementary Table 12. Transcription factors expressed in early and late RPCs, transcriptionally targeted by and/or predicted to interact with LHX2 are among those with high pioneer potential

Differentially expressed genes (DEG) set identified by pairwise comparison of RNA-Seq from E14 and P2 RPCs from *Chx10-CreGFP* mice. Protein Interaction quantitation (PIQ) were computed on P2 RPCs chromatin accessibility profiles, compared to E14. Non-redundant IDs are listed, with the highest RNA-Seq reads counts and PIQ score for any given transcription factor. DEG genes versus non DEG genes (FPKM ≥ 5) matching transcription factor s with predicted pioneer activity: p-val (t-test, mean pioneer value) > 0.05. Stage specific genes versus shared genes matching transcription factors with predicted pioneer activity: p-val (t-test, mean pioneer value) > 0.05. Transcription factor motifs occurrences estimated on age-matched LHX2 ChIP-Seq (Supplementary Figure 7A,B). For any given transcription factor, the corresponding variation in gene expression (RNA-Seq) and local accessibility of the closest related cis-regulatory site (ATAC-Seq) is reported from *Lhx2* cKO age-matched conditions. Indicated in

bold (blue/red font for E14/P2) are the transcription factors exhibiting variations in average footprints counts upon LHX2 loss of function.

Supplementary References

1. Van Helden, J., Regulatory sequence analysis tools. *Nucleic Acids Res* **31**, 3593-3596 (2003).
2. Castro-Mondragon JA., Jaeger S., Thieffry D., Thomas-Chollier M, Van Helden J. RSAT matrix-clustering: dynamic exploration and redundancy reduction of transcription factor binding motif collections. *Nucleic Acid Res*, **45**:13 e119 (2017)
3. Trapnell, C. et al., Differential gene and transcript expression analysis of RNA-seq experiments with TopHat and Cufflinks. Nat Protoc **7**, 562-578 (2012).
4. Heinz, S. *et al.*, Simple combinations of lineage-determining transcription factors prime cis-regulatory elements required for macrophage and B cell identities. *Mol Cell* **38**, 576-589 (2010).
5. De Melo, J., Zibetti C., Clark B., Hwang W., Miranda-Angulo A., Qian J., Blackshaw S., Lhx2 Is an Essential Factor for Retinal Gliogenesis and Notch Signaling. *J Neurosci* **36**, 2391-2405 (2016).
6. De Melo, J., Clark B, Venkataraman A., Shiau F., Zibetti C., Blackshaw S. Ldb1- and Rnf12-dependent regulation of Lhx2 controls the relative balance between neurogenesis and gliogenesis in the retina. Development **145**, 1-10 (2018).
7. Tetreault N., Champagne M.P., Bernier G. The LIM homeodomain transcription factor Lhx2 is required to specify the retina field and synergistically cooperates with Pax6 for Six6 transactivation. Dev. Biol. 327, 541-550 (2009).
