# Supplementary material for: Epigenomic profiling of retinal progenitors reveals LHX2 is required for developmental regulation of open chromatin
Source: Commun Biol. 2019 Apr 25;2:142. doi: 10.1038/s42003-019-0375-9 (PMC6484012; doi:10.1038/s42003-019-0375-9)
Supplement: Supplementary file 3 — Description of additional supplementary items [file 42003_2019_375_MOESM3_ESM.docx]

**Description of additional supplementary items**

**Supplementary Data 1. Curated list of genes that show enriched or specific expression pattern in individual cell types in developing and mature retina**

Genes that show expression in one or more cell types in developing and mature retina, along with embryonic anterodorsal hypothalamus, are listed. AC=amacrine cells, BC=bipolar cells, HC=horizontal cells, MG=Müller glia, RGC=retinal ganglion cells. If a gene has been reported to show expression in the cell type or region indicated, this is indicated with a “1”, or a “0” if expression is low/absent. Sources for this information are indicated by PubMed IDs. The criteria used to measure gene expression and score selective expression is also indicated. The RPC-enriched genes sets were derived from the RNA-Seq profiling of flow sorted GFP-positive and GFP-negative cell fractions in this study.

**Supplementary Data 2. *Lhx2-*dependent, differentially expressed gene sets**

**(2.1-2.3)** Differentially expressed genes (DEG) identified from pairwise comparison of flow sorted E14 and P2 RNA-Seq profiles from *Chx10-CreGFP* mice (E14/P2 GFP+; E14 GFP+/-; P2 GFP+/-) and the related variation in E14 and P2 *Lhx2* cKO conditions are reported. **(2.4)** Retinal Gene IDs populating the ontologies displayed in Fig. 2C-F and Supplementary Figure 5H, associated to proximal LHX2 age-matched ChIP-Seq peaks and/or showing transcriptional dependence on LHX2, are also reported.

**Supplementary Data 3. Paired variations in sequencing coverage at LHX2 cis-regulatory sites across datasets and footprinting analysis of altered transcriptional networks in *Lhx2* cKO**

Genomic accessibility by ATAC-Seq, promoter/enhancer occupancy by age-matched LHX2 and H3K27Ac ChIP-Seq and expression levels by RNA-Seq are reported in control and *Lhx2* cKO conditions. The nearest promoters, ChIP-Seq peaks were assigned to, were defined by extending TSS regions 5000 bp upstream, 1000 bp downstream for up to 1000000 bp max extension. Enhancers were defined by H3K27ac coverage. Transcription factors exhibiting footprints variations at promoters and nearby enhancers in *Lhx2* cKO are indicated.

**Supplementary Data 4. Metadata Description for GEO repository.**
